# Quadruplex–Duplex Junction: A High‐Affinity Binding Site for Indoloquinoline Ligands

**DOI:** 10.1002/chem.202003540

**Published:** 2020-11-16

**Authors:** Yoanes Maria Vianney, Pit Preckwinkel, Swantje Mohr, Klaus Weisz

**Affiliations:** ^1^ Institute of Biochemistry Universität Greifswald Felix-Hausdorff-Str. 4 17487 Greifswald Germany

**Keywords:** calorimetry, DNA structures, G-quadruplexes, NMR spectroscopy

## Abstract

A parallel quadruplex derived from the *Myc* promoter sequence was extended by a stem‐loop duplex at either its 5′‐ or 3′‐terminus to mimic a quadruplex–duplex (Q–D) junction as a potential genomic target. High‐resolution structures of the hybrids demonstrate continuous stacking of the duplex on the quadruplex core without significant perturbations. An indoloquinoline ligand carrying an aminoalkyl side chain was shown to bind the Q–D hybrids with a very high affinity in the order *K*_a_≈10^7^ m ^−1^ irrespective of the duplex location at the quadruplex 3′‐ or 5′‐end. NMR chemical shift perturbations identified the tetrad face of the Q–D junction as specific binding site for the ligand. However, calorimetric analyses revealed significant differences in the thermodynamic profiles upon binding to hybrids with either a duplex extension at the quadruplex 3′‐ or 5′‐terminus. A large enthalpic gain and considerable hydrophobic effects are accompanied by the binding of one ligand to the 3′‐Q–D junction, whereas non‐hydrophobic entropic contributions favor binding with formation of a 2:1 ligand‐quadruplex complex in case of the 5′‐Q–D hybrid.

## Graphical abstract

**An indoloquinoline‐based** G4 ligand binds with very high affinity at the quadruplex–duplex junction of a parallel G‐quadruplex featuring a stem‐loop duplex extension. Thermodynamic profiles and modes of binding differ for hybrids with duplex extensions at either the 5′‐ or 3′‐face of the G‐quadruplex.

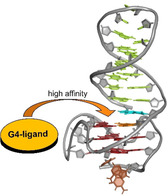

## Introduction

Sequences with four runs of G‐nucleotides can fold into G‐quadruplexes (G4s) composed of stacked G‐quartets and further stabilized by the coordination of monovalent cations.^[1]^ G4 forming sequences have been found throughout the genome, with frequent occurrences in telomeres and promoter regions of human oncogenes such as c*‐Myc*, *c‐Kit*, and *KRAS*.[^2^, ^3^, ^4^] Intramolecular G‐quadruplexes with their four G‐columns connected by loops show highly diverse topologies. This polymorphism is reflected in different types of loops but may also include discontinuous G‐tracts.[^5^, ^6^, ^7^, ^8^, ^9^, ^10^] In general, various G4 topologies in the genome may be specifically targeted by high‐affinity ligands for novel pharmaceutical approaches, but quadruplex topologies can also be rationally designed for use in an increasing number of technological applications, for example, as aptamers.[^11^, ^12^, ^13^, ^14^]

DNA junctions are important elements in cellular maintenance processes.[^15^, ^16^, ^17^] Given that G‐quadruplex forming sequences in gene promoter regions originate from duplex DNA,^[18]^ the presence of quadruplex–duplex (Q–D) motifs seems obvious. Q–D hybrid structures have already been reported 25 year ago^[19]^ and several variants have since been developed by placing the duplex forming sequence at different internal or external positions of the G4.[^11^, ^20^, ^21^, ^22^] In fact, bioinformatic studies have revealed the abundance of such Q–D hybrid structures in the human genome.^[23]^ Consequently, Q–D junctions may be considered hotspots of a druggable region in guiding a ligand to bind at the G‐quadruplex structure with high selectivity while retaining high affinity. Accordingly, hybrid ligands designed by simply joining known G4 ligands with duplex minor groove binders have been proposed for targeting such type of junctions.[^13^, ^24^]

**Figure 1:**
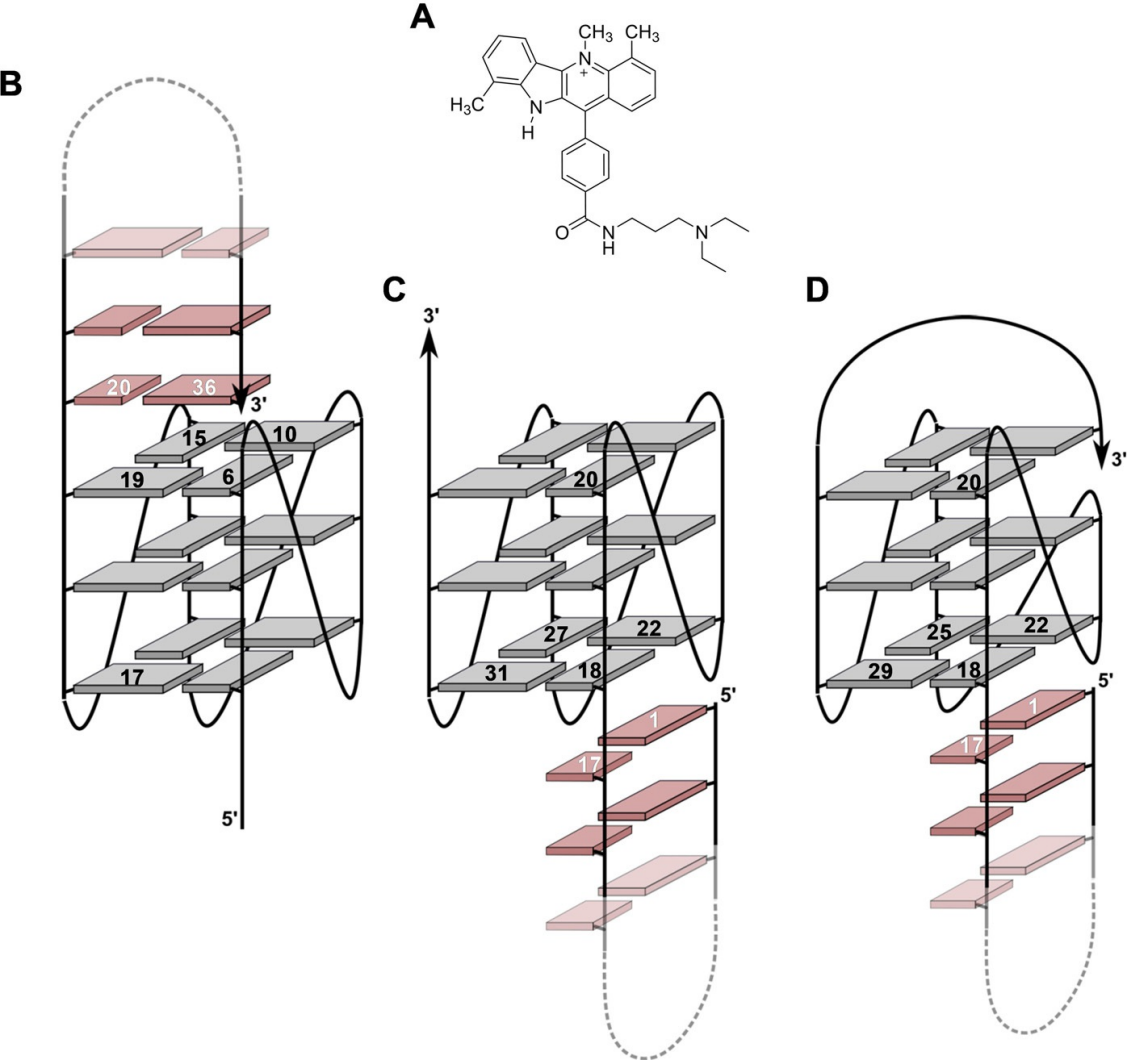
Structure of the PIQ ligand (A). Schematic representation of Q–D hybrids *Myc‐dup3* (B), *Myc*‐*dup5* (C), and *Myc3l‐dup5* (D) with numbering of residues at the Q–D junction; G‐tetrad guanines and base pairs of the duplex hairpin extension are indicated by grey and red rectangles, respectively.

One major challenge for the design of a ligand as specific G4 probe or drug is the selectivity for a quadruplex with respect to other DNA secondary structures but also to a unique quadruplex topology.[^25^, ^26^, ^27^] It is also generally understood that an increase in selectivity, for example, through discrimination of a quadruplex versus duplex species, will often be associated with a decrease in affinity.[^28^, ^29^] In previous studies we developed a ligand termed PIQ based on a phenyl‐substituted indoloquinoline heterocyclic ring system (Figure 1 A).^[30]^ This ligand showed high selectivity and affinity towards the parallel *Myc* quadruplex. While stacking on the outer G‐tetrad of the *Myc* G4 is favored and associated with the formation of a binding pocket through short overhang sequences, the PIQ ligand was shown to be tightly sandwiched between the 3′‐faces of two quadruplexes in a 1:2 complex when binding a 3′‐truncated *Myc* sequence.^[31]^ However, affinity towards duplex DNA is also observed and promoted by its positively charged aminoalkyl substituent, albeit to a small extent.^[30]^

In the present paper, a Q–D junction was designed through a duplex stem‐loop extension at either the quadruplex 5′‐ or 3′‐terminus (Figure 1). Detailed structural and thermodynamic analysis was employed to characterize the binding behavior of a PIQ ligand as a typical G4 binder. Using the major *Myc* quadruplex but also a modified and extended version with a snap‐back loop architecture at its 3′‐end as G4 module within the hybrids, the results suggest that PIQ binds selectively and with high affinity to the Q–D junction. However, thermodynamic binding profiles noticeably differ for binding at a junction adjacent to the 5′‐ or 3′‐face of the quadruplex G‐core.

## Results and Discussion

### Circular dichroism spectra

The present design of a quadruplex–duplex hybrid is based on the well‐studied parallel G4 derived from the promoter region of the *c‐Myc* oncogene.^[32]^ A long overhang sequence at either the 5′‐ or 3′‐terminus of the G4 core is designed to form a duplex stem‐loop structure with a CG base pair adjacent to the 5′‐ or 3′‐outer tetrad of the quadruplex, respectively (Figure 1). In contrast to base pair formation within loop regions of the G‐core, the hybrids *Myc‐dup5* and *Myc‐dup3* are expected to exhibit increased flexibility at the Q–D junction because only a single attachment at one end of the double‐helical stem‐loop structure links the duplex to the quadruplex motif.

**Figure 2:**
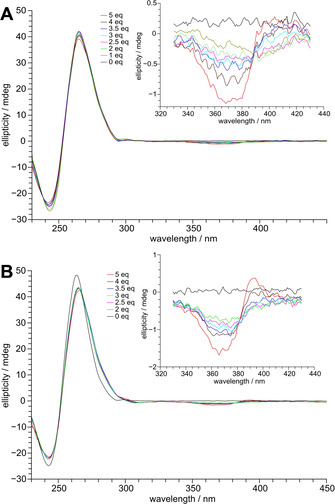
CD spectra of A) *Myc‐dup3* and B) *Myc‐dup5* following titration with PIQ (0–5 equivalents); the inset shows induced CD effects at the ligand absorption.

CD spectra of the Q–D hybrids are shown in Figure 2. They exhibit negative and positive maxima at around 240 and 265 nm typical for a parallel quadruplex topology with stacked G‐tetrads of the same polarity and with all‐*anti* glycosidic torsion angles for the G‐core residues. Because a B‐type duplex shares similar CD signatures, the additional presence of a duplex stem‐loop structure remains mostly hidden. However, ellipticities in *Myc‐dup3* with a double‐helical domain at the G4 3′‐face are somewhat reduced compared to *Myc‐dup5* but also native *Myc* with only short overhangs (Figure S1).^[30]^ On the other hand, the duplex stem‐loop structure at the 5′‐ or 3′‐terminus does not seem to noticeably impact the *Myc* parallel topology.

Upon titrating the PIQ ligand, no major changes were observed in the CD spectra below 300 nm, implying that the topology of both quadruplexes was retained after PIQ binding. Also, the appearance of induced circular dichroism (ICD) effects around the long wavelength absorption maximum of the ligand at 376 nm demonstrates binding of the achiral ligand to the chiral environment of the DNA. It should be noted that small changes in ellipticity at λ<300 nm during addition of ligand may indicate small quadruplex conformational adjustments but may also be attributed to ICD effects through short wavelength absorptions of bound PIQ.

ICD effects as a function of increasing ligand concentration give first hints of PIQ binding to the Q–D hybrids. A broad negative ICD band is observed at the start of titration, in line with ligand stacking on an outer tetrad, but is typically also observed for intercalative binding of a planar ligand to duplex DNA.^[33]^ In subsequent titration steps the amplitude of the ICD band increases at its lower but decreases at its higher wavelength side, becoming even positive for *Myc‐dup5* with a five‐fold excess of ligand (Figure 2). Such a behavior indicates the gradual onset of an additional binding process characterized by a single positive ICD overlapping the initial band at longer wavelengths. Interestingly, a corresponding ICD is seen for the duplex‐free *Myc* G4 known to bind PIQ at its exposed outer tetrads (Figure S1). Alternatively, a developing bisignate ICD due to exciton couplings between bound ligands in close proximity and with negative and positive amplitudes at lower and higher wavelengths, respectively, may superimpose on the initial CD band.

### Melting experiments

Bound ligand is expected to affect the stability of DNA as reflected in changes of its melting temperature *T*_m_, which are conveniently determined by temperature‐dependent UV and CD experiments. For a separate determination of duplex and quadruplex melting, wavelengths used for the measurements are of particular importance. In UV melting experiments, a hyperchromicity at *λ*=260 nm is expected upon the transition from duplex to single strand whereas the absorbance at *λ*=295 nm is most sensitive to quadruplex melting with associated hypochromic effects. However, for quadruplex melting studies in the presence of ligand, temperature‐dependent CD experiments were performed to avoid complications due to interferences from ligand absorption below 300 nm but also due to closely similar quadruplex and duplex melting temperatures (see below). With a maximum of its positive band for the parallel G4 topology, a decreased CD signal at 265 nm mostly indicates quadruplex unfolding because ellipticities at this wavelength for the B‐type duplex hairpin extensions are less pronounced.

For lowering the high melting temperature of the *Myc* quadruplex, melting experiments were initially performed in a low‐salt buffer with 10 mm K^+^. Under these conditions, duplex and quadruplex domains in *Myc‐dup5* and *Myc‐dup3* melt in two well‐separated individual transitions. Melting temperatures for the duplex motifs are 11 and 20 °C below melting of the corresponding quadruplex domains (Table S2 and Figure S2). As expected from the close structural similarity only flanked by a long single‐stranded overhang at either the 5′‐ or 3′‐terminus after duplex melting, the UV‐derived *T*_m_ value for the G4 subunit in *Myc‐dup5* was found to be 67 °C and thus only slightly higher by 2 °C when compared to *Myc‐dup3*. For a validation of these quadruplex‐ and duplex‐specific results, additional DSC experiments were performed. Deconvolution of the two transitions yielded melting temperatures *T*_m_ in full agreement with those obtained from the temperature‐dependent absorbances at 295 and 260 nm (Table S2 and Figure S3).

As a consequence of premature duplex melting under the low salt conditions, evaluation of a ligand‐induced G4 thermal stabilization suffers from the lack of a defined Q–D junction. We therefore changed the buffer solution from 10 mm K^+^ to 120 mm Na^+^, expecting that a higher ionic strength will stabilize the duplex whereas the replacement of potassium by sodium ions will likely destabilize the quadruplex domain without affecting its topology, as demonstrated previously.[^1^, ^30^, ^34^] Indeed, as shown by UV and CD melting experiments, relative stabilities of duplex and quadruplex domains change under the new buffer conditions, resulting in a duplex melting more than 10 °C above G4 melting for both hybrids (Table 1 and Figure S2). Also, *T*_m_ values for the G4 units in the Na^+^ buffer with the flanking duplexes intact differ by 8 °C and suggest more stabilizing duplex‐G4 interactions at the 5′‐ when compared to the 3′‐outer tetrad.

**Table 1:** Melting temperatures *T*_m_ for the Q–D hybrids without and with addition of 1 equivalent of PIQ in 100 mm NaCl, 20 mm sodium phosphate buffer, pH 7.^[a]^

Q–D hybrid	*T*_m_ (duplex) [°C]		*T*_m_ (quadruplex) [°C]	
	w/o PIQ^[b]^	with 1 equiv PIQ^[b]^	w/o PIQ^[c]^	with 1 equiv PIQ^[c]^
*Myc‐dup3*	63.6±0.6	70.8±0.3	44.7±1.8	68.2±1.4
*Myc‐dup5*	67.8±0.6	73.5±1.0	53.2±0.6	69.7±1.2
*Myc3l‐dup5*	69.5±0.5	76.1±0.3	66.3±0.4	73.8±0.4

[a] Averages with standard deviation from three independent measurements except for the quadruplex melting of *Myc3l‐dup5* with and without PIQ which was determined in duplicate. [b] *T*_m_ data from UV melting experiments. [c] *T*_m_ data from CD melting experiments.

With the addition of 1 equivalent of ligand in Na^+^ buffer, similar *T*_m_ values determined by the temperature dependence of UV absorbances for the duplex and of CD ellipticities for the quadruplex seem to merge at about 70 °C and suggest a single yet rather broad melting transition for both hybrid‐ligand complexes given different methods and experimental uncertainties (Table 1). With the duplex domain only moderately stabilized due to ligand binding by Δ*T*_m_≤7 °C, the quadruplex shows a significant stabilization by 17 and 23 °C for *Myc‐dup5* and *Myc‐dup3*, respectively. The higher ligand‐induced thermal stabilization of the *Myc‐dup3* quadruplex in Na^+^ buffer compensates for its noticeably lower intrinsic stability when compared to *Myc‐dup5*. Also, the increase in melting temperatures for both duplex and quadruplex domains with the addition of ligand in a 1:1 molar ratio points to strong ligand interactions at the Q–D interface.

### Thermodynamics of ligand binding to the Q–D hybrids

**Figure 3:**
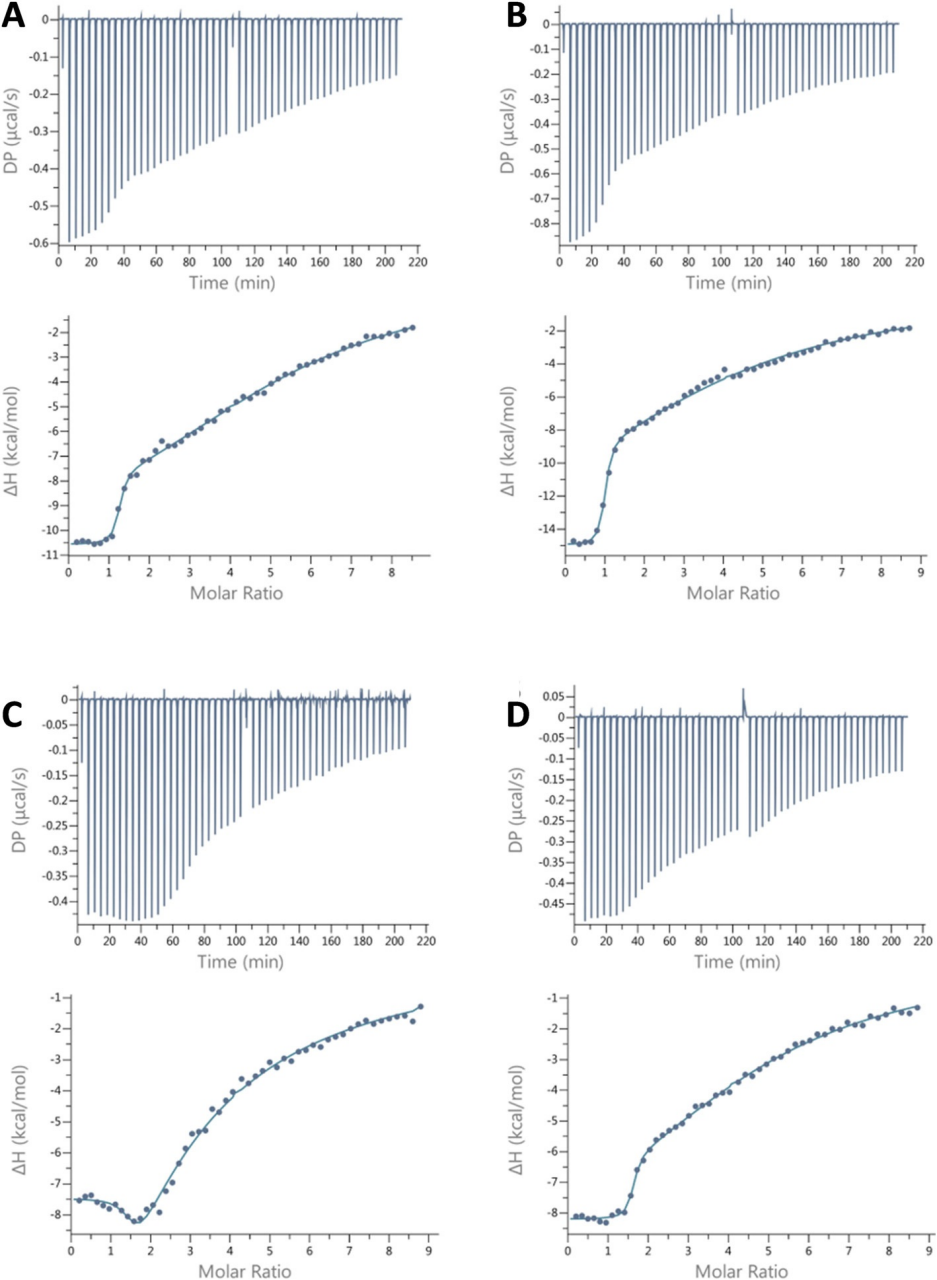
Representative ITC thermograms for the binding of PIQ at 120 mm K^+^ to A) *Myc‐dup3* at 40 °C, B) *Myc‐dup3* at 50 °C, C) *Myc‐dup5* at 40 °C, and D) *Myc3l‐dup5* at 40 °C. The upper and lower panels show the heat burst for every injection step and the dilution‐corrected heat versus the molar ratio, respectively.

Isothermal titration calorimetry was used to determine the thermodynamic profile of PIQ binding to the Q–D hybrids. Experiments were performed by titrating the ligand into a 120 mm K^+^ buffer solution of the oligonucleotides. Thermograms obtained after integration of the power output for each injection and corrections for the heats of dilution are shown in Figure 3.

The titration curve for *Myc‐dup3* acquired at *T=*40 °C and thus close to physiological temperatures, point to the presence of a single high‐affinity binding site with the release of heat upon ligand binding (Figure 3 A). A gradual return to baseline at high ligand‐to‐DNA molar ratios suggests additional weaker binding events and the presence of multiple nonequivalent binding sites. Potential sites include the Q–D junction, the 5′‐outer tetrad with its short overhang, and the duplex domain, but unspecific binding, for example, through electrostatic interactions with the negatively charged DNA backbone, should also be considered. Employing a two‐site model for curve fitting, excellent and reproducible fits were obtained for the high‐affinity binding associated with a very high exothermicity, a stoichiometry of 1, and an association constant of *K*_a_=1.6×10^7^ m ^−1^ (Table 2). Initial 1:1 complex formation is clearly separated from ensuing binding processes of weaker affinity. Given that more than one subsequent binding event of lower affinity are anticipated to superimpose at later titrations, a second set of thermodynamic parameters for low affinity binding sites through curve fitting was not evaluated in more detail.

**Table 2:** ITC‐derived thermodynamic parameters for the binding of PIQ to the Q–D hybrids in the presence of 120 mm K^+^ at 40 °C.^[a]^

Q–D hybrid	N	*K*_a_ [m ^−1^]	Δ*H*°_fit_ [kcal mol^−1^]^[b]^	Δ*G*° [kcal mol^−1^]^[c]^	Δ*H*°_es_ [kcal mol^−1^]^[b]^	−*TΔS*° [kcal mol^−1^]^[c]^
*Myc‐dup3*	1.2±0.1	(1.6±0.4)×10^7^	−10.7±0.1	−10.3±0.2	−11.9±0.5	1.5±0.5
*Myc‐dup3* ^[d]^	1.1±0.2	(1.5±0.5)×10^7^	−15.0±0.8	−10.6±0.2	−14.8±0.2	4.3±0.3
*Myc‐dup5* ^[e]^	n.d.	n.d.	n.d.	n.d.	−6.7±0.3	n.d.
*Myc3l‐dup5*	1.5±0.1	(1.6±0.2)×10^7^	−8.2±0.1	−10.3±0.1	−7.3±0.2	−3.0±0.2

[a] Average values with standard deviations from three independent measurements; only values for the high‐affinity binding are given. [b] Δ*H*°_fit_ and Δ*H*°_es_ denote standard molar enthalpy changes determined from curve fitting and from an excess‐site method, respectively. [c] From Δ*G*°=−*RT*ln*K*_a_ and −*TΔS*°=Δ*G*°−Δ*H*°_es_. [d] At 50 °C. [e] Isotherm could not be reliably fitted with a two‐site model.

To get more insight into potential binding sites, it is instructive to look at closely related DNA‐ligand associations. Recently, an association constant *K*_a_ of 2×10^6^ m ^−1^ and a binding enthalpy Δ*H*° of −6.3 kcal mol^−1^ at 40 °C has been determined for PIQ binding to the outer tetrads of the *Myc* quadruplex lacking a duplex extension.^[35]^ To also assess binding to the duplex domain, we performed ITC titrations of the separate duplex stem‐loop structure with the ligand (Figure S4). Here, corresponding binding isotherms indicate a released heat of <5 kcal mol^−1^ and rather weak binding with estimates of *K*_a_≤10^4^ m ^−1^. These results strongly suggest that the Q–D interface in *Myc‐dup3* constitutes the binding site of highest affinity. Binding here is also associated with a very high exothermicity not found in either free quadruplex or hairpin duplex.

Isotherms for *Myc‐dup5* at 40 °C exhibit a shallow minimum following a plateau region at initial titration steps and a gradual return to baseline with excess of ligand. It is immediately apparent that such a heat profile indicates the presence of more than two calorimetrically distinct association processes with multiple binding sites at the G4 hybrids. The superposition of more than two binding events during the entire course of titration compromises the extraction of binding parameters through curve fitting for *Myc‐dup5* and even restricts the determination of reliable thermodynamic parameters for the site of highest affinity. To nevertheless obtain a more accurate binding enthalpy for the latter, we employed an excess‐site method to yield a Δ*H*° of about −7 kcal mol^−1^ at 40 °C (Figure S5). An excess of DNA in this protocol ensures that added ligand is completely bound to high‐affinity sites for every injection step and that the area under each power output directly reflects the molar binding enthalpy following normalization.^[36]^

### Introducing a Q–D hybrid variant with a 3′‐snap‐back loop

Due to the limitations in analyzing thermograms of *Myc‐dup5*, a variant termed *Myc3l‐dup5* was designed and tested for ligand binding (Figure 1). *Myc3l‐dup5* is based on the quadruplex *Myc3l* that carries an additional 3′‐extension together with a central two‐nucleotide deletion and was previously shown to fold into a snap‐back loop structure. Because the snap‐back loop spans the quadruplex 3′‐face and effectively prevents the ligand from binding at its 3′‐tetrad,[^5^, ^37^] elimination of an additional putative binding site should provide for a better resolved thermogram. On the other hand, this variant is expected to closely mimic PIQ binding at the *Myc‐dup5* 5′‐face with its duplex extension because binding opposite the snap‐back loop should be essentially unperturbed (see below). UV and CD melting data of *Myc3l‐dup5* are also summarized in Table 1.

When titrating the *Myc3l‐dup5* hybrid with the PIQ ligand, a well‐defined first binding event with reproducible thermodynamic parameters and an apparent stoichiometry >1 could be extracted (Figure 3 D). With a *K*_a_ of 1.6×10^7^ m ^−1^ it matches with the high‐affinity binding of *Myc‐dup3* and again suggests binding at the interface of quadruplex and duplex domains. PIQ binding at the 5′‐face with no noticeable perturbations by the opposite snap‐back loop structure is also strongly suggested by the close binding enthalpies Δ*H*° of about −7 kcal mol^−1^ extracted by an excess‐site method for the high‐affinity binding for both *Myc‐dup5* and *Myc3l‐dup5*.

Of note, although featuring the same association constant for a proposed binding at the Q–D interface, thermodynamic profiles of PIQ binding are strikingly different for *Myc3l‐dup5* and *Myc‐dup3*. A significantly more favorable binding enthalpy for *Myc‐dup3* is counteracted by a slight loss in entropy whereas a less exothermic binding to *Myc3l‐dup5* is associated with a favorable change in entropy at 40 °C to give the same Gibbs free energies (Table 2). Additional ITC titrations for *Myc‐dup3* at 50 °C show no significant change in binding constant but a noticeably more negative binding enthalpy of about −15 kcal mol^−1^ (Figure 3 B, Table 2). These results suggest extensive enthalpy‐entropy compensation effects with a negative molar heat capacity Δ*C*_p_° exceeding entropic changes Δ*S*°, as frequently observed for small molecules binding to a biomolecular receptor.^[38]^

To determine Δ*C*_p_° for high‐affinity binding, binding enthalpies were determined at 20, 30, 40, and 50 °C by an excess‐site method and plotted over temperature to give a Δ*C*_p_° of −221 cal mol^−1^ K^−1^ and −101 cal mol^−1^ K^−1^ for *Myc‐dup3* and *Myc3l‐dup5*, respectively (Table 3, Figures S6 and S7). Whereas Δ*C*_p_° upon binding *Myc3l‐dup5* is close in magnitude to changes in heat capacity effects as obtained for other quadruplex‐ligand interactions,[^39^, ^40^] Δ*C*_p_° for *Myc‐dup3* binding is highly negative, in particular when compared to a Δ*C*_p_°=−67 cal mol^−1^ K^−1^ determined for PIQ binding to the *Myc* G4 lacking a duplex extension.^[35]^ However, a larger change in heat capacity of about −330 cal mol^−1^ K^−1^ was reported for minor groove binding of Hoechst 33258 to duplex DNA.^[41]^

**Table 3:** Temperature‐dependent binding enthalpies Δ*H*°,^[a]^ heat capacities Δ*C*_p_°, and hydrophobic contributions to the Gibbs total free energy Δ*G*°_hyd_ for PIQ binding to the Q–D hybrids.

Q–D Hybrid	Δ*H*°_es,293 K_	Δ*H*°_es,303 K_	Δ*H*°_es,313 K_	Δ*H*°_es,323 K_	Δ*C*_p_°	Δ*G*°_hyd_^[b]^
	[kcal mol^−^]	[kcal mol^−^]	[kcal mol^−^]	[kcal mol^−^]	[cal mol^−^ K^−^]	[kcal mol^−^]
*Myc‐dup3*	−8.1±0.5	−10.1±0.2	−11.9±0.5	−14.8±0.2	−221±19	−17.7
*Myc3l‐dup5*	−5.4±0.1	−6.2±0.2	−7.3±0.2	−8.5±0.2	−101±8	−8.1

[a] Average values for the high‐affinity binding with standard deviations from three independent measurements. [b] From the relationship Δ*G*°_hyd_=80⋅Δ*C*_p_°.

In general, negative Δ*C*_p_° indicates a reduced solvent‐accessible surface area associated with hydrophobic effects by the release of water at the nonpolar solute‐solvent interface. Based on solvent‐transfer experiments of liquid hydrocarbons, the semi‐empirical relationship Δ*G*°_hyd_=80⋅Δ*C*_p_° links molar heat capacity changes with the hydrophobic driving force of association Δ*G*°_hyd_.^[42]^ It should be noted that this correlation strictly applies to temperatures near 20 °C and assumes a Δ*C*_p_° that exclusively results from hydrophobic effects. Regardless of such uncertainties, favorable contributions from hydrophobic effects are clearly major contributors for ligand binding to *Myc3l‐dup5*. On the other hand, hydrophobic interactions are suggested to play an even more dominant role for ligand binding to *Myc‐dup3* with its 3′‐duplex extension, exhibiting a Δ*G*°_hyd_ twice as large as determined for *Myc3l‐dup5*. With hydrophobic effects being mostly entropic in nature, a less favorable change in total entropy as found for ligand binding to *Myc‐dup3* is quite unexpected (Table 2). Apparently, residual entropic contributions differ considerably for these two hybrids and point to more significant reductions in conformational entropy and flexibility in complexes of *Myc‐dup3*.

### NMR solution structure of Q–D hybrids

**Figure 4:**
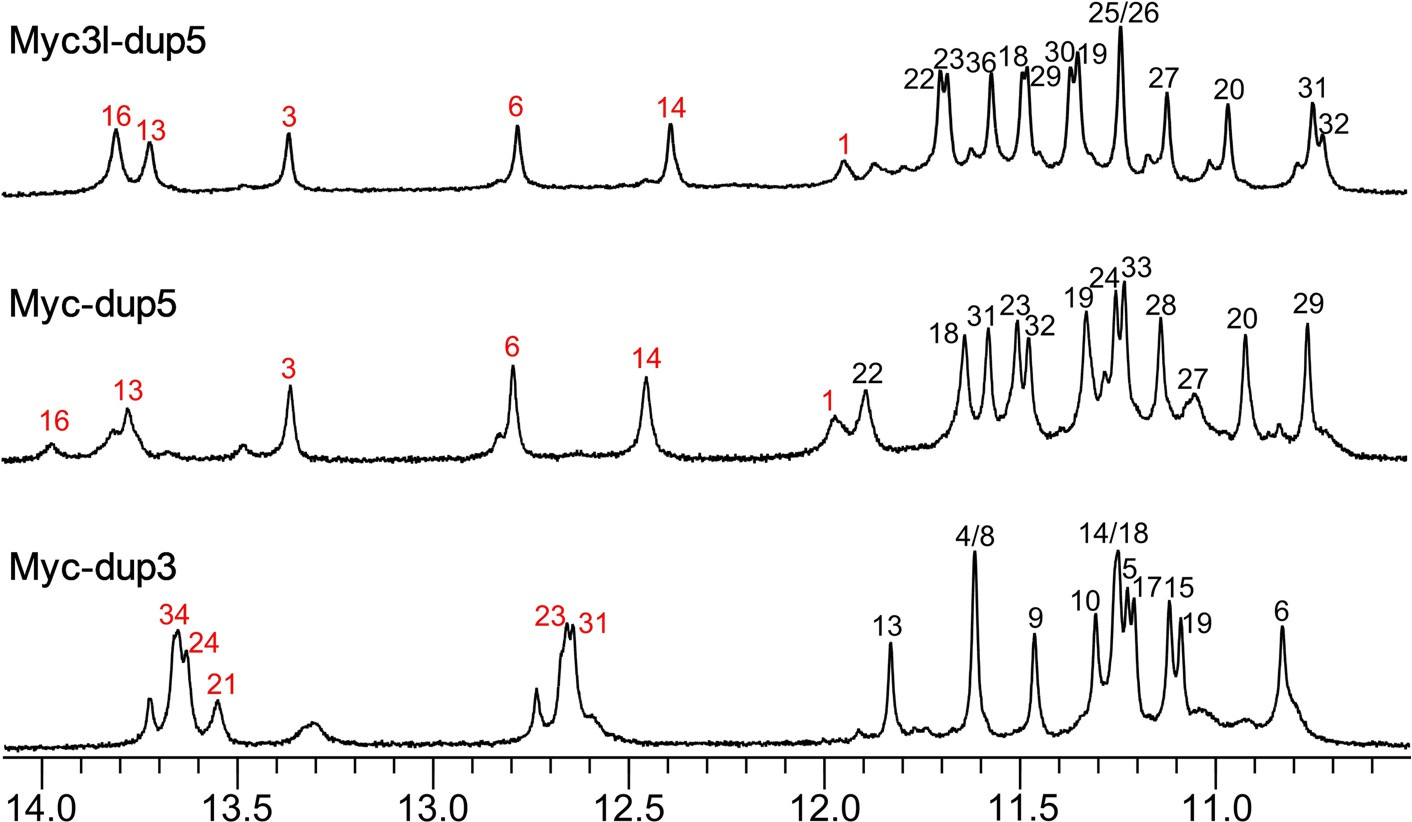
Imino proton spectral region of *Myc‐dup3*, *Myc‐dup5*, and *Myc3l‐dup5*; assigned resonances from the G‐core and Watson–Crick stem‐loop duplex are indicated by black and red numbers, respectively.

Initially all three Q–D hybrids, namely *Myc‐dup3*, *Myc‐dup5*, and *Myc3l‐dup5* were structurally characterized in detail by NMR analysis. ^1^H NMR spectra for all three sequences revealed some low‐intensity signals of minor species in the imino proton spectral region but these did not hamper proton assignments of the predominant hybrid structure (Figure 4). Thus, 12 major Hoogsteen imino proton resonances in the 10.5–12 ppm region indicate an intact three‐layered G‐quadruplex core for *Myc‐dup3* and *Myc‐dup5*. For *Myc3l‐dup5* an additional slow‐exchanging imino signal of a guanine base within the snap‐back loop is observed, in line with its participation in a capping base triad, as reported previously.[^5^, ^37^] The detection of Watson–Crick imino resonances between 12–14 ppm demonstrate complementary base pairing in the stem‐loop structure for the 3′‐ and 5′‐flanking sequences. All iminos involved in Watson–Crick hydrogen bonding of the duplex region have been successfully assigned except for the imino signal of the AT base pair adjacent to the T_3_ hairpin loop and, in case of *Myc‐dup3*, for the imino resonance of the GC base pair at the Q–D junction, likely due to its fast exchange with solvent.

In general, an almost complete resonance assignment following standard strategies based on the analysis of DQF‐COSY, ^1^H‐^13^C HSQC, and 2D NOESY experiments was achieved (Figures S8–S10). Intranucleotide and sequential H6/H8‐H1′ contacts in NOESY spectra of all three sequences suggest no major structural perturbations of the quadruplex through the formed duplex stem‐loop extension. In fact, H6/H8‐H1′ contacts enable a continuous NOE walk from G17 at the 5′‐end of the last G‐tract of the quadruplex until the 3′‐terminal guanosine G36 of the duplex in *Myc‐dup3* and from the 5′‐terminal G1 until G20 at the 3′‐end of the first G‐tract in *Myc‐dup5* and *Myc3l‐dup5*. Several NOE connectivities involving base and sugar protons between residues in the G‐tetrad and the following base pair were observed that define the geometry of the Q–D junction (Table S3). These include a G36 H8‐G6 H1 contact in *Myc‐dup3*, a G22 H8‐G1 H1 contact in *Myc‐dup5*, and G1 H8‐G22/G25 H1 contacts in *Myc3l‐dup5* (Figures S8–S10). Various non‐sequential contacts connect C17 of the tetrad‐flanking base pair in *Myc‐dup5* and *Myc3l‐dup5* with G31 and G29 of the adjacent tetrad, respectively, and point to the cytosine being more directed towards the G‐core when compared to *Myc‐dup3*.

**Figure 5:**
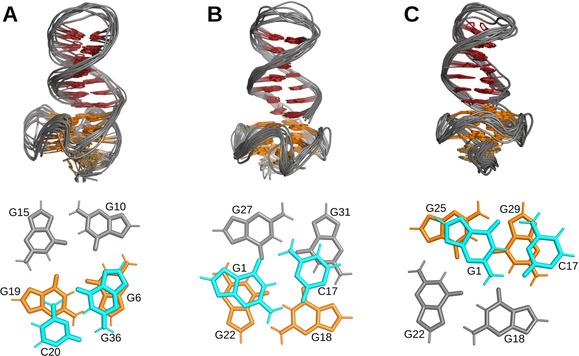
Top: Superposition of ten lowest energy structures of A) *Myc‐dup3*, B) *Myc‐dup5*, and C) *Myc3l‐dup5*. Only the backbone is shown for loop residues. Bottom: Top view of the Q–D junction with stacking interactions between the first duplex CG base pair and the adjacent quadruplex outer tetrad.

Based on NMR‐derived distance and torsion angle restraints, structures were calculated for all three Q–D hybrids (Figure 5, Table S4). Extending from the parallel G‐quadruplex domain, the long 3′‐ and 5′‐flanking sequences form a B‐type hairpin structure with a T_3_ loop. Residues in the short overhangs at the opposite quadruplex face, that is, G2 and A3 in *Myc‐dup3* as well as A35 in *Myc‐dup5*, cap the tetrad plane. On the other hand, the 3′‐overhang in *Myc3l‐dup5* forms a snap‐back loop that is additionally stabilized by a G32‐A34‐G35 triad above the 3′‐tetrad in seven out of the ten low‐energy structures.

It should be noted that the present Q–D hybrids differ from previously reported Q–D architectures that harbor the duplex domain within a G4 loop or form a double helix through complementary 5′‐ and 3′‐overhang sequences.[^11^, ^20^, ^22^] With only one strand linked to the G‐core, the duplex is expected to exhibit increased flexibility. Nevertheless, with 12–17 NOE‐derived distance restraints between base pair and adjacent G‐tetrad for each hybrid, the geometry of the Q–D junction is well defined. Interestingly, different stacking patterns between the G⋅C Watson–Crick base pair and the neighboring quadruplex tetrad are apparent in the hybrid structures (Figure 5). The Q–D junction in *Myc‐dup3* is similar to constructs with a double‐helical lateral loop (PDB ID 2M8Z and 2M90).^[20]^ It shows nearly maximum stacking between terminal G36 of the duplex and G6 of the 3′‐tetrad with cytosine C20 only poorly stacked on the 5′‐sequential guanine G19 (Figure 5 A). For the hybrids with the duplex at the 5′‐outer tetrad, the CG base pair at the junction is turned towards the G‐quadruplex core. As a result, the base‐paired cytosine exhibits no significant stacking interactions with the 3′‐linked guanine of the G‐tetrad but is shifted to partially stack on a guanine at the adjacent G‐tetrad edge (Figure 5, B and C). Whereas the base pair is centrally stacked on the G‐tetrad in *Myc‐dup5*, the stacking pattern for the *Myc3l‐dup5* hybrid is reminiscent of the previously reported Q–D construct incorporating a duplex with a G⋅A pair to form a diagonal loop (PDB ID 2M91).^[20]^

### NMR structural studies on PIQ binding to *Myc‐dup3*

**Figure 6:**
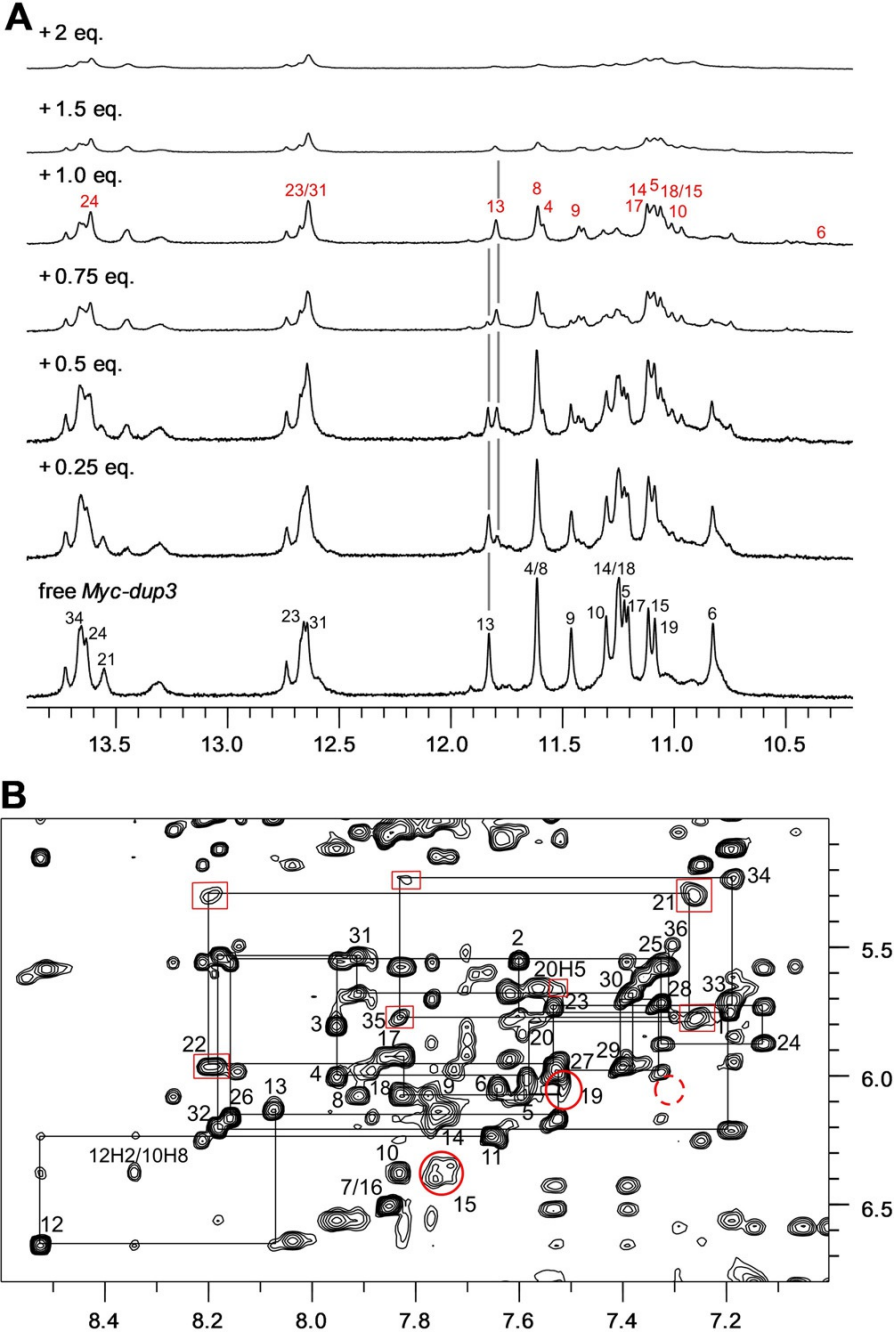
NMR spectra of *Myc‐dup3* (0.53 mm) at 20 °C. A) Imino proton spectral region upon addition of the PIQ ligand. Imino proton resonances are assigned to G residues in free *Myc‐dup3* and in the ligand‐hybrid complex by black and red numbers, respectively. Vertical lines indicate the position of G13 H1 signals with their opposing change in intensity for free and complexed *Myc‐dup3*. B) H6/8(ω_2_)‐H1′(ω_1_) spectral region of a 2D NOESY spectrum (300 ms mixing time) in the presence of one equivalent of PIQ. A continuous walk by intranucleotide and sequential NOEs can be traced from G17 of the fourth G‐run of the quadruplex until the terminal G36 residue of the duplex. Crosspeaks broadened with respect to the free hybrid spectra are framed by red boxes and red circles for duplex and quadruplex protons, respectively. The dashed red circle indicates the position of a missing contact between residue G36 and G6 at the junction.

Titrating the *Myc‐dup3* hybrid with the PIQ ligand is accompanied by the appearance of a new set of G4 imino proton resonances in the 10–12 ppm spectral region (Figure 6 A). The latter gradually increase up to a 1:1 molar ratio where free Q–D hybrid resonances have essentially disappeared and imino groups of the newly formed species predominate. Adding ligand in excess, more significant signal broadening indicates exchange processes between complexes at intermediate timescales.

At a 1:0.5 hybrid‐to‐ligand molar ratio the spectra point to the coexistence of equally populated free and ligand‐bound DNA in slow exchange, which is clearly also demonstrated by two sets of crosspeaks with about equal intensity in a corresponding 2D NOESY spectrum (Figure S11). Unfortunately, ROESY experiments on such samples failed to reveal correlations between the two species, in line with very slow exchange rates (data not shown). Therefore, proton assignments for the complex, making use of standard methodologies for a conventional parallel quadruplex and a B‐type duplex, were based on samples with one equivalent of added ligand (Figure 6 B). Uninterrupted NOE walks in the H6/8‐H1′ spectral region can be traced from the 3′‐terminal G36 of the duplex extension to G17 in the fourth G‐tract of the quadruplex core and also from the 5′‐overhang to G6 in the first G‐column. Additional NOE connectivities exist between A12 within the propeller loop and guanines in the second and third G‐tract. Proton assignments of the complex were completed based on guanine H8‐imino and imino‐imino NOE contacts of the quadruplex core (Figure S12).

**Figure 7:**
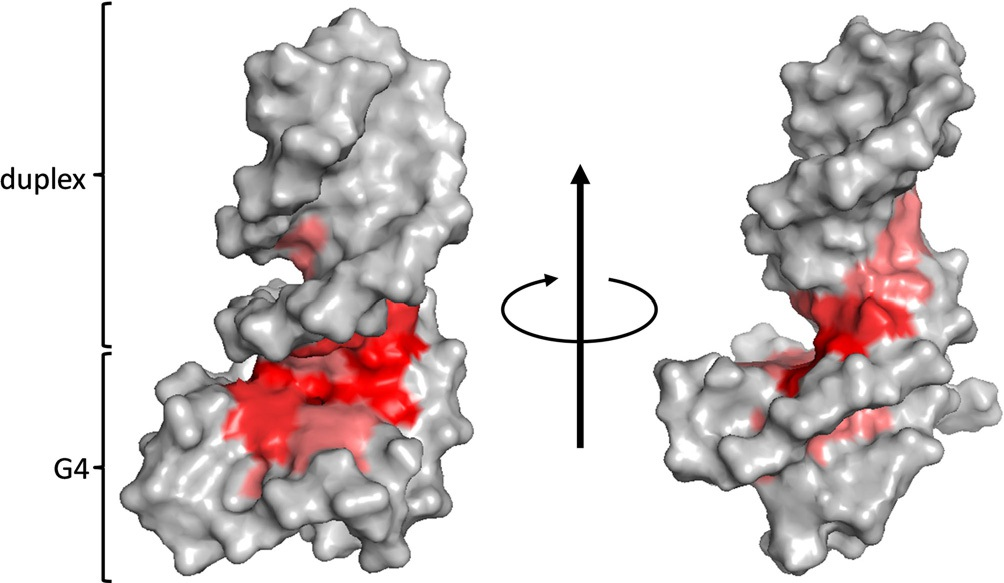
*Myc‐dup3* proton chemical shift perturbations after addition of 1 equivalent of PIQ mapped with red color of variable intensity on a surface model of the hybrid. View onto the 3′‐tetrad of the quadruplex–duplex junction (left) and rotation around the *z*‐axis with view into the duplex minor groove (right). For a more detailed compilation of chemical shift data see Figure S13 and Table S5 and S6.

A chemical shift footprint was constructed by plotting ^1^H chemical shift changes through ligand binding and these were also mapped on a surface model of the *Myc‐dup3* structure by red color of varying intensity (Figure S13, Figure 7). Inspection of the data reveals substantial perturbations of imino and H8 protons for residues located within the 3′‐tetrad except for G15. Although being less affected, the H8‐H1′ intranucleotide NOE crosspeak of the latter seems considerably broadened, possibly due to environments changing at intermediate frequencies (Figure 6 B). Likewise, residues of the duplex domain located close to the Q–D junction, that is, C20, T21, and A35, exhibit more significant chemical shift changes. Inspection of the surface model with mapped perturbations immediately identifies the Q–D junction at the 3′‐tetrad as major binding site for the ligand with putative additional interactions of the PIQ sidechain within the duplex minor groove in a 1:1 complex (Figure 7). It should be noted, however, that duplex chemical shift changes for non‐junction residues may also result from conformational readjustments after ligand binding.

Signal broadening upon the addition of ligand in excess to give a 1:2 hybrid‐to‐ligand molar ratio severely hampered resonance assignments of the quadruplex domain. However, ^1^H resonances of the duplex remained reasonably sharp, which enabled their complete assignment by following walks along H6/H8‐H1′ NOE contacts (Figure S14A). Conspicuously, no significant chemical shift differences for assigned duplex proton resonances in *Myc‐dup3* spectra were observed when going from a 1:1 to a 1:2 hybrid‐to‐ligand molar ratio. This also includes residues near the junction and suggests that the ligand neither binds at the duplex nor at the Q–D junction in subsequent titration steps.

Two crosspeaks between G2 and A3 H1′ of the 5′‐overhang and imino protons of the 5′‐tetrad were observed in the free hybrid and persisted after addition of 1 equivalent of ligand (Figure S14B). However, these two crosspeaks disappeared or considerably shifted after the addition of 2 PIQ equivalents, suggesting signal broadening due to exchange processes and/or conformational readjustments as a result of ligand binding at the 5′‐tetrad. Supported by the ITC data and in line with ICD effects in the CD spectra (see Figures 2 and S1), the present NMR data demonstrate a single high‐affinity binding site at the Q–D junction followed by the occupation of the 5′‐outer tetrad with ligand in excess.

For fixing the bound ligand in a defined orientation, additional assignments of ligand protons and the observation of intermolecular NOE contacts are indispensable. NOESY spectra of the *Myc‐dup3* hybrid in the presence of equimolar amounts of ligand exhibit new crosspeaks of a resonance at 10.15 ppm to protons of the 3′‐outer tetrad; that is, to G6 H8, G19 H8, and G15 H1 (Figure S12). The deshielded proton at 10.15 ppm likely identifies the indole NH of the indoloquinoline ring system rather than the amide NH of the PIQ sidechain, suggesting stacking of the indoloquinoline on the 3′‐tetrad. However, NOE contacts to both G6 H8 and G19 H8 for a single PIQ proton in a unique orientation seems questionable based on interproton distances within the planar tetrad. In fact, various exchange peaks for ligand aromatic signals also including the resonance at 10.15 ppm point to different orientations of bound ligand. Also, no clear NOE contacts are observed between any ligand proton and the duplex domain of *Myc‐dup3*. However, some broadening of H8‐H1′ crosspeaks for C20, T21, A22, and A35 residues of the stem‐loop structure as well as for G15 and G19 of the 3′‐tetrad may indicate intermediate exchange between complexes with different ligand orientation, corroborating the absence of a single well‐defined complex structure (Figure 6 B).

### NMR structural studies on PIQ binding to *Myc‐dup5* and *Myc3l‐dup5*

**Figure 8:**
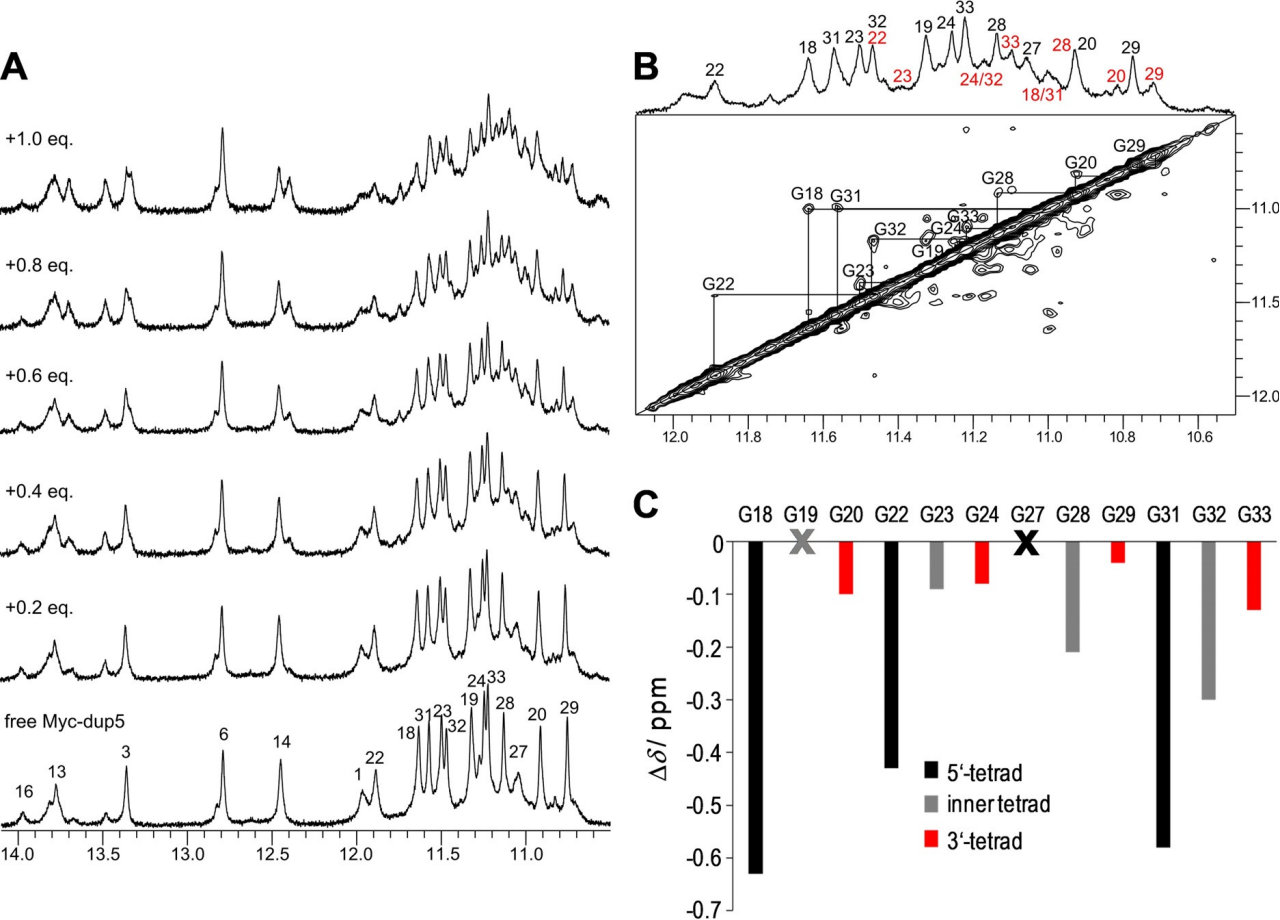
A) Imino proton spectral region of *Myc‐dup5* (0.5 mm) titrated with PIQ at 20 °C. B) 1D and ROESY spectrum showing the G4 imino proton spectral region of *Myc‐dup5* in the presence of 0.6 equivalent of PIQ at 20 °C. Exchange crosspeaks connect guanine imino resonances of free and complexed species. Imino proton resonances are assigned to G residues in free *Myc‐dup5* and in the ligand‐hybrid complex by black and red numbers, respectively. C) Imino chemical shift differences of G4 residues between complexed (with 0.6 equiv. of PIQ) and free *Myc‐dup5*. Imino resonances of G19 and G27 could not be unambiguously assigned.

Upon titrating *Myc‐dup5* and *Myc3l‐dup5* with ligand, new resonances gradually appeared in analogy to *Myc‐dup3* in the G4 imino proton spectral region between 10 and 12 ppm (Figures 8 A and S15A). These indicate slowly exchanging species, that is, coexisting free and ligand bound DNA hybrids. Notably, however, signals of free *Myc‐dup5* and *Myc3l‐dup5* do not vanish but rather persist after the addition of one equivalent of PIQ with roughly equal populations of free and bound species based on imino signal intensities. Such a behavior contrasts sharply with high‐affinity binding in a 1:1 stoichiometry to *Myc‐dup3* and seems to confirm ITC experiments that consistently indicated higher stoichiometries of *N*>1 for strong ligand binding to *Myc3l‐dup5*.

Other differences in binding to quadruplexes with 5′‐ and 3′‐duplex extensions are apparent when assessing their kinetic behavior. After the addition of 0.5–0.6 equivalent of ligand to *Myc‐dup5* or *Myc3l‐dup5*, exchange peaks of G4 imino protons can be observed in ROESY experiments as a consequence of faster exchange rates between free and bound species (Figures 8 B and S15B). Although resonance assignments for the complex are mostly precluded due to the extensive crowding and overlap of signals in 2D NOESY spectra, exchange peaks allow assignments for most imino protons in the ligand‐DNA complex based on assigned resonances of the free hybrid. It should be noted that additional exchange crosspeaks of low intensity in the ROESY spectra point to the presence of minor complex species and therefore only strong exchange peaks indicative of a major complex were used in the construction of chemical shift footprints.

Large upfield shifts are observed for G imino protons at the 5′‐outer G4 tetrad in *Myc‐dup5* (Figure 8 C, Figure S16). Such a pattern clearly demonstrates preferential PIQ binding at the 5′‐tetrad and thus at the Q–D junction. On the other hand, minor chemical shift changes for protons in the inner and 3′‐tetrad may mostly be due to some conformational adjustments following ligand binding. Additional confirmation of high‐affinity binding sites at the quadruplex 5′‐face with its duplex extension comes from *Myc3l‐dup5* (Figure S15C). As for *Myc‐dup5*, chemical shifts of imino protons in the 5′‐tetrad experienced most significant perturbations when compared to protons of the inner and 3′‐tetrad. Also, a similar chemical shift perturbation pattern was found for *Myc‐dup5* and *Myc3l‐dup5* upon the addition of less than 1 equivalent of ligand, confirming similar binding characteristics of both hybrids at their 5′‐Q–D junction and only a minor impact of the 3′‐snap‐back loop on binding at the opposite Q–D interface.

### Ligand binding and thermodynamic profiles

Association constants for PIQ binding to all Q–D hybrids exceed affinities observed for PIQ binding to the *Myc* quadruplex with only short three‐nucleotide flanking sequences or to the corresponding *Myc*‐derived G4 with a 3′‐snap‐back loop by nearly one order of magnitude.[^35^, ^37^] Chemical shift perturbations upon ligand binding clearly identify the Q–D junction as high‐affinity binding site. However, Q–D junctions at the 5′‐ and 3′‐face of the quadruplex seem to vary considerably in their stability. Thus, lower melting of the quadruplex domain of free *Myc‐dup3* in a sodium‐containing buffer, that is, with the duplex extension mostly intact (Table 1), points to higher flexibilities at the junction, as also indicated by a non‐observable G36⋅C20 base pair imino resonance likely due to fraying effects (see above).

In addition to different quadruplex–duplex interactions at the junction of the hybrids, the 5′‐outer tetrad of the *Myc* G4 is more hydrophobic and more accessible to additional stacking than the 3′‐outer tetrad, often resulting in a noticeable selectivity of ligand binding to *Myc* for one of its two G4 faces.^[43]^ Likewise, altered binding stoichiometries and binding modes are also suggested by the structural and thermodynamic studies for the 3′‐ and 5′‐junctions. Given the large association constant *K*_a_>10^7^ m ^−1^ as determined by ITC for the high‐affinity binding (Table 2), the presence of equally populated free and ligand bound *Myc‐dup5* and *Myc3l‐dup5* after the addition of one PIQ equivalent seems only compatible with the cooperative binding of two molecules of ligand or the binding of a ligand dimer to the Q–D hybrids. As a result, only one binding event is detected by the ITC experiment, although the ligand environment and the strength of ligand‐hybrid interactions are anticipated to differ for two PIQ ligands bound to the same G4 receptor. Interestingly, Gibbs free energies for PIQ binding are identical for the 3′‐ and 5′‐duplex hybrids, yet thermodynamic profiles with enthalpic and entropic contributions differ significantly in line with noticeable differences in binding. Although structural details of a 2:1 PIQ‐hybrid complex are lacking, binding at or close to the junction is demonstrated by the NMR structural studies. Owing to its planar surface area of only moderate size with the non‐fused phenyl substituent rotated out of the indoloquinoline plane, two PIQ molecules can be conceived to stack in a side by side fashion on top of the 5′‐outer tetrad of *Myc‐dup5* and *Myc3l‐dup5* with additional stabilizing interactions provided by the ligand sidechain.

Stacking of the indoloquinoline heterocyclic ring system on the outer G4 tetrad of the Q–D junction is consistent with large upfield shifts of guanine imino protons located within the tetrad plane. Given that high‐resolution structures of all the free hybrids show continuous stacking of the duplex extension onto the G‐quadruplex, partial insertion of the ligand between base pair and G‐tetrad can be assumed. In fact, the loss of a 2D NOESY crosspeak between 3′‐terminal G36 H8 and G6 H1 linking the duplex and quadruplex domain in the *Myc‐dup3*–ligand complex corroborates the assumption of a ligand (partially) sandwiched between quadruplex and duplex extension (Figure 6 B). To date there is no precedent for a three‐dimensional structure with a ligand intercalated between a base pair and G‐tetrad at a Q–D junction. Interestingly, however, the G4 ligand 360A has recently been reported to intercalate between GC‐GC and GA‐GA duplexes within a tetrahelical topology with a flexible central cavity. Binding tightens the structure to create GCGC and GAGA tetrads while increasing the distance between adjacent bases to accommodate the inserted ligand.^[44]^

Given high‐affinity binding at the Q–D junction for quadruplexes with either 3′‐ or 5′‐duplex extensions, the absence or presence of exchange crosspeaks between free and ligand bound Q–D hybrids in ROESY spectra of *Myc‐dup3* and *Myc‐dup5* deserves further discussion. In contrast to *Myc‐dup5*, exchange rates are slow compared to the mixing time of the ROESY experiment for *Myc‐dup3*. Slower ligand dissociation kinetics can be attributed to stronger ligand‐DNA interactions, which are also reflected by the more negative binding enthalpy as determined by ITC experiments. Given a more flexible junction in free *Myc‐dup3* (see above), the considerable enthalpic gain through ligand binding may be attributed to stronger interactions with the ligand, accompanied by a stiffening of the junction in line with less favorable non‐hydrophobic contributions to the binding entropy. It would be tempting to correlate the different thermodynamic binding profiles and associated hydrophobic effects with particular structural characteristics, but different binding stoichiometries and the lack of a well‐defined ligand binding mode in *Myc‐dup3* and *Myc‐dup5*/*Myc3l‐dup5* exclude a more detailed assessment of structure‐stability relationships.

## Conclusions

In addition to the targeting of individual quadruplex architectures, the recognition of a Q–D junction can expand possibilities for a selective DNA targeting by low molecular weight ligands. Such an approach is based on the idea that Q–D junctions are potential hotspots and widely occurring structural elements in the genome. In fact, Q–D junctions may be formed during biological processes associated with the unwinding of a putative G‐quadruplex forming sequence either internally as part of a long self‐complementary quadruplex loop or externally at the transition from quadruplex to the canonical B‐type double‐helical structure.

Previous strategies have employed hybrid ligands composed of a quadruplex specific ligand linked with a duplex minor groove binder.[^13^, ^24^] However, Q–D junctions may by themselves constitute high‐affinity binding sites for known G4 specific ligands, favoring the junction over stacking at exposed outer tetrads of an individual G4 structure as shown here for an indoloquinoline‐based ligand. Although not directly clear from their three‐dimensional structures, junctions at opposite faces of the G‐quadruplex core exhibit pronounced differences in thermal stabilities affected by interactions between the quadruplex and duplex domains. Likewise, thermodynamic profiles for ligand binding are noticeably different for double‐helical 3′‐ and 5′‐extensions of the parallel G4. More favorable binding enthalpies, more hydrophobic contributions to the Gibbs free energy through a more negative change in molar heat capacity, and less favorable entropic changes when binding to a Q–D junction at the G4 3′‐face are expected to considerably change relative affinities by altering temperatures. It may also hint at a future drug design to selectively address enthalpic and entropic contributions for discriminating between binding at the two quadruplex faces. Together with their high affinity towards appropriate ligands, Q–D junctions may thus constitute targets with a high potential for therapeutic interventions.

## Experimental Section

### Materials and sample preparation

PIQ was prepared as described previously and its concentration was determined spectrophotometrically by using a molar extinction coefficient *ϵ*_376_=22 227 L mol^−1^ cm^−1^.^[30]^ DNA oligonucleotides were purchased from TIB MOLBIOL (Berlin, Germany) and further purified by a potassium acetate‐ethanol precipitation. DNA concentrations were determined spectrophotometrically by measuring absorbances A_260_ at 80 °C in water using molar extinction coefficients as supplied by the manufacturer. Prior to usage, oligonucleotides were dried, redissolved in buffer, heated to 90 °C and annealed by slowly cooling to RT. Sequences of oligonucleotides are given in Table S1.

### UV/Vis melting experiments

The Q–D hybrid was dissolved in 1.5 mL of either 10 mm potassium phosphate buffer, pH 7, or 100 mm NaCl, 20 mm sodium phosphate buffer, pH 7.0. UV/Vis experiments were performed with a Jasco V‐650 spectrophotometer (Jasco, Tokyo, Japan) equipped with a Peltier thermostat. For measurements of the duplex *T*_m_, the final DNA concentration was 2 μm and absorbance was recorded at *λ*=260 nm as a function of temperature (10–90 °C). Data were acquired with a bandwidth of 1 nm and a heating rate of 0.2 °C min^−1^. For measurements of the quadruplex *T*_m_, the final DNA concentration was 5 μm and absorbance was recorded at *λ*=295 nm between 10 and 90 °C with parameters as given for duplex melting. The melting temperature was determined by the first derivative of the melting curve. For the melting of complexes, the ligand was added up to a 1:1 molar ratio.

### CD spectroscopy

CD spectra were measured at 20 °C on the Q–D hybrids (5 μm) in 100 mm KCl, 20 mm potassium phosphate buffer, pH 7.0. A concentrated PIQ solution in DMSO was added up to a 5:1 ligand‐to‐DNA molar ratio. The DMSO concentration of the mixture was always <1 %. All measurements were performed with a Jasco J‐810 spectropolarimeter equipped with a Peltier thermostat (Jasco, Tokyo, Japan). Spectra were recorded for solutions in 1 cm quartz cuvettes from 230 to 450 nm with a bandwidth of 1 nm, a scanning speed of 50 nm min^−1^, a response time of 4 s, and five accumulations. Prior to measurements, the quadruplex‐ligand mixtures were stirred for 10 min to ensure equilibration.

For the determination of melting temperatures, the Q–D hybrid (5 μm) in the absence or presence of 1 equivalent of PIQ was redissolved in 100 mm NaCl, 20 mm sodium phosphate buffer, pH 7.0. Ellipticities were recorded at *λ*=265 nm between 20 and 95 °C with a bandwidth of 1 nm and a heating rate of 0.2 °C min^−1^. Melting temperatures were determined by the first derivative of the melting curve.

### Isothermal titration calorimetry

ITC experiments were performed with a Microcal PEAQ ITC microcalorimeter (Malvern Instruments, United Kingdom) employing a reference power of 4 μcal s^−1^. Oligonucleotides and the PIQ ligand were each dissolved in 100 mm KCl, 20 mm potassium phosphate buffer, pH 7.0, supplemented with 5 % DMSO. The PIQ solution (400 μm) was titrated to 20 μm of oligonucleotide with a total of 2×26 injections of 1.5 μL each, an injection duration of 3 s, and a spacing between injections of 240 s. The first injection (0.4 μL) was rejected during the fitting process. Excess‐site titrations were performed to determine model‐independent binding enthalpies directly from averages of peak integrals of the power outputs. For each of the 12 titration steps, 3 μL of ligand solution (200 μm) with a 6 s injection duration were titrated to the oligonucleotide solution (100 μm) with a spacing between injections of 300 s. The first injection volume (0.4 μL) was not included in the calculation of binding enthalpies. Measurements at a temperature range of 20–50 °C were performed to determine the change of heat capacity upon binding. All experiments were blank‐ and concentration‐corrected. For data analysis, the MicroCal PEAQ‐ITC analysis software was used.

### NMR spectroscopy

NMR spectra were acquired with a Bruker Avance 600 MHz spectrometer equipped with an inverse ^1^H/^13^C/^15^N/^19^F quadruple resonance cryoprobehead and *z*‐field gradients. Data were processed in TopSpin 4.0.7 and assigned in CcpNmr V2.^[45]^ Oligonucleotides were dissolved in 10 mm potassium phosphate buffer, pH 7.0. Upon titration with a PIQ solution in [D_6_]DMSO, the final DMSO concentration with addition of 2 equivalents of ligand was below 5 %. Proton chemical shifts were referenced through the water chemical shift taking into account its temperature dependence at pH 7 and carbon chemical shifts were referenced to DSS through an indirect referencing method. Residual HOD in D_2_O solutions for DQF‐COSY experiments was suppressed by presaturation. A WATERGATE w5 sequence was generally used for water suppression in solutions of 90 % H_2_O/10 % D_2_O except for ^1^H‐^13^C HSQC experiments employing a 3–9–19 pulse sequence. The latter experiments were acquired with a spectral width of 7500 Hz in the F1 dimension, 4 K×500 data points, and a 1 s recycle delay. Zero‐filling gave a 4 K×1 K data matrix that was multiplied with a sine‐bell squared window function in both dimensions. Homonuclear 2D spectra were typically recorded with 2 K×1 K data points with a relaxation delay of 2 s. Prior to Fourier transformation, FID data were zero‐filled to give a final 4 K×1 K data matrix and processed with a sine‐bell squared window function in both dimensions. 2D NOESY spectra were acquired with mixing times from 80 to 300 ms and ROESY spectra were recorded with a mixing time of 80 ms.

### Structure calculation and molecular modeling

Starting structures (100) of lowest energy were selected out of 200 structures generated by a simulated annealing protocol in XPLOR‐NIH 2.52.^[46]^ Distance restraints were obtained from crosspeak intensities in NOESY spectra. Distances were categorized as follows: 2.9±1.1 Å for strong crosspeaks, 4.0±1.5 Å for crosspeaks of medium intensity, 5.5±1.5 Å for weak crosspeaks, and 6.0±1.5 Å for very weak crosspeaks. For overlapped peaks, the distance was set to 5.0±2.0 Å. All χ torsion angles were set as either anti (170–310°) or syn (25–95°) while all sugar puckers were set to the south domain (pseudorotational angle 144–180°). Planarity restraints were employed for tetrads and base pairs.

Restrained simulated annealing was performed using AMBER16 with the parmbsc force field and OL15 modifications.^[47]^ In vacuo refinement was done for 100 starting structures to yield 20 converged structures by initially equilibrating the system at 300 K for 5 ps followed by heating the system to 1000 K during 10 ps and keeping the temperature for the next 30 ps. The system was cooled to 100 K within 45 ps and finally to 0 K within 10 ps.

Restraint energies for simulated annealing in AMBER were 40 kcal mol^−1^ Å^−2^ for NOE based distance restraints, 50 kcal mol^−1^ Å^−2^ for hydrogen‐bond based distance restraints, 200 kcal mol^−1^ rad^−2^ for dihedral angle restraints, and 30 kcal mol^−1^ Å^−2^ for tetrad and base pair planarity restraints.

Refinement in water was done by initially neutralizing the system with potassium ions and placing two potassium ions in the inner core of the quadruplex flanked by two tetrad layers. The system was soaked with TIP3P water in a 10 Å truncated octahedral box, initially minimized with 500 steps of steepest descent minimization followed by another 500 steps of conjugate gradient minimization. Both quadruplex and duplex domains were fixed with a force constant of 25 kcal mol^−1^ Å^−2^. The system was heated under constant volume from 100 to 300 K in 10 ps. The system was further equilibrated under a constant pressure of 1 atm with energy restraints decreasing to 5, 4, 3, 2, 1, and 0.5 kcal mol^−1^ Å^−2^. A final simulation was done at 1 atm and 300 K for 4 ns. Snapshots were taken for every 1 ps, the trajectory was averaged for the last 500 ps and shortly minimized in vacuo to obtain 10 lowest‐energy structures.

Chemical shift perturbations upon ligand binding were mapped on a surface model of the hybrids by coloring residues depending on chemical shift differences of their imino, base and H1′ protons. Each proton was assigned a value based on the maximum change in chemical shift Δ*δ*_max_ observed for resonances of the same type that was set to 100 %. This was followed by averaging and grouping into three classes with decreasing averaged perturbations that were assigned a red color of decreasing intensity.

### Accession codes

Atomic coordinates and lists of chemical shifts have been deposited for *Myc‐dup3* (PDB ID 6ZL2, BMRB ID 34524), *Myc‐dup5* (PDB ID 6ZL9, BMRB ID 34525), and *Myc3l‐dup5* (PDB ID 6ZTE, BMRB ID 34533).

## Conflict of interest

The authors declare no conflict of interest.

## Supporting information

As a service to our authors and readers, this journal provides supporting information supplied by the authors. Such materials are peer reviewed and may be re‐organized for online delivery, but are not copy‐edited or typeset. Technical support issues arising from supporting information (other than missing files) should be addressed to the authors.

Supplementary
