# Supplementary material for: Quadruplex–Duplex Junction: A High‐Affinity Binding Site for Indoloquinoline Ligands
Source: Chemistry. 2020 Nov 16;26(70):16910–22. doi: 10.1002/chem.202003540 (PMC7756412; doi:10.1002/chem.202003540)
Supplement: Supplementary file 1 — Supplementary [file CHEM-26-16910-s001.pdf]

Chemistry–A European Journal

Supporting Information

Quadruplex–Duplex Junction: A High-Affinity Binding Site for Indoloquinoline Ligands

Yoanes Maria Vianney, Pit Preckwinkel, Swantje Mohr, and Klaus Weisz*[a]

Table of Contents

Table S1: Sequences of oligonucleotides S2

Table S2: Melting temperatures of *Myc-dup3* and *Myc-dup5* S2

Table S3: NOE-based distance restraints S3

Table S4: NMR restraints and structural statistics S5

Table S5: ^{1}H chemical shifts of free *Myc-dup3* S6

Table S6: ^{1}H chemical shifts of *Myc-dup3* with one equivalent PIQ S7

Figure S1: CD spectra of *Myc* upon PIQ addition S9

Figure S2: Optical melting curves of *Myc-dup3*, *Myc-dup5*, and *Myc3l-dup5* S10

Figure S3: DSC melting curves of *Myc-dup3* and *Myc-dup5* S11

Figure S4: ITC thermograms of PIQ binding to duplex hairpins S12

Figure S5: Excess-site ITC thermogram of PIQ titrated to *Myc-dup5* S12

Figure S6: Excess-site ITC titrations of PIQ to *Myc-dup3* and *Myc3l-dup5* S13

Figure S7: Plot of $\Delta H°$ over temperature for PIQ binding to the Q-D hybrids S14

Figure S8: NOESY spectral regions of *Myc-dup3* S15

Figure S9: NOESY spectral regions of *Myc-dup5* S16

Figure S10: NOESY spectral regions of *Myc3l-dup5* S17

Figure S11: NOESY spectral region of *Myc-dup3* with 0.5 eq. of PIQ S18

Figure S12: NOESY spectral regions of *Myc-dup3* with 1 eq. of PIQ S19

Figure S13: ^{1}H chemical shift footprints of *Myc-dup3* S20

Figure S14: NOESY spectral regions of *Myc-dup3* with addition of PIQ S21

Figure S15: Spectra and chemical shift footprints of *Myc3l-dup5* upon PIQ titration S22

Figure S16: Surface model of *Myc-dup5* with chemical shift perturbations S23

Table S1. Sequences of oligonucleotides; duplex hairpin domains within the Q-D hybrids are underlined.

name	sequence
Myc	5'-TGA GGG T GGG TA GGG T GGG TAA
Myc-dup3	5'-TGA GGG T GGG TA GGG T GGG CTAGTCA TTT TGACTAG-3'
Myc-dup5	5'-GATCAGT TTT ACTGATC GGG T GGG TA GGG T GGG TA-3'
Myc3l-dup5	5'-GATCAGT TTT ACTGATC GGG T GG T GGG T GGG GAAGG-3'
Dup3	5'-CTAGTCA TTT TGACTAG-3'
Dup5	5'-GATCAGT TTT ACTGATC-3'

Table S2. UV- and DSC-derived melting temperatures T_m of *Myc-dup3* and *Myc-dup5* in 10 mM potassium phosphate buffer, pH 7.[a]

	T_m from UV-vis (°C)		T_m from DSC (°C)	
Myc-dup3	duplex	45.1 ± 0.3	1st transition	45.9 ± 0.1
	quadruplex	65.1 ± 0.2	2nd transition	64.5 ± 0.4
Myc-dup5	duplex	56.1 ± 0.3	1st transition	56.5 ± 0.1
	quadruplex	66.9 ± 0.5	2nd transition	66.5 ± 0.1

[a] Averages with standard deviations from three independent experiments.

Table S3. NOE-based distance restraints for residues at the quadruplex-duplex junction in *Myc-dup3*, *Myc-dup5*, and *Myc3l-dup5*.

	Myc-dup3	
duplex	quadruplex	distance (Å)
C20-H5	G19-H8	4.0 ± 1.5
C20-H5	G19-H2’	5.5 ± 1.5
C20-H5	G19-H2”	4.0 ± 1.5
C20-H5	G19-H1’	4.0 ± 1.5
C20-H5	G19-H3’	5.5 ± 1.5
C20-H6	G19-H1’	4.0 ± 1.5
C20-H6	G19-H2’	4.0 ± 1.5
C20-H6	G19-H2”	2.9 ± 1.1
C20-H6	G19-H3’	5.5 ± 1.5
G36-H8	G6-H1’	5.5 ± 1.5
G36-H1’	G6-H1’	4.0 ± 1.5
G36-H2”	G6-H1’	2.9 ± 1.1
G36-H1’	G6-H2’	4.0 ± 1.5
G36-H1’	G6-H2”	4.0 ± 1.5
G36-H8	G6-H1	4.0 ± 1.5
G36-H2’	G6-H1	6.0 ± 1.5
G36-H2”	G6-H1	6.0 ± 1.5
	Myc-dup5	
duplex	quadruplex	distance (Å)
C17-H1’	G18-H8	4.0 ± 1.5
C17-H3’	G18-H8	4.0 ± 1.5
C17-H6	G18-H8	5.5 ± 1.5
C17-H5	G31-H8	6.0 ± 1.5
G1-H1’	G22-H8	5.5 ± 1.5
C17-H2’	G31-H1	5.5 ± 1.5

C17-H2”	G31-H1	6.0 ± 1.5
C17-H6	G31-H1	5.5 ± 1.5
G1-H1’	G22-H1	4.0 ± 1.5
G1-H2”	G22-H1	6.0 ± 1.5
G1-H1	G22-H8	5.5 ± 1.5
G1-H1	G31-H1	4.0 ± 1.5

Myc3l-dup5

duplex	quadruplex	distance (Å)
G1-H8	G25-H8	5.5 ± 1.5
C17-H1’	G18-H8	4.0 ± 1.5
C17-H2’	G18-H8	4.0 ± 1.5
C17-H2”	G18-H8	2.9 ± 1.1
C17-H3’	G18-H8	5.5 ± 1.5
C17-H6	G18-H8	5.5 ± 1.5
C17-H6	G29-H1’	5.5 ± 1.5
G1-H2’	G22-H1	5.5 ± 1.5
G1-H8	G22-H1	6.0 ± 1.5
G1-H1’	G22-H1	4.0 ± 1.5
G1-H2”	G22-H1	5.5 ± 1.5
G1-H8	G25-H1	6.0 ± 1.5
C17-H6	G29-H1	6.0 ± 1.5
C17-H1’	G29-H1	4.0 ± 1.5

Table S4. NMR restraints and structural statistics of calculated structures.

	Myc-dup3	*Myc-dup5*	*Myc3l-dup5*
NOE distance restraints:			
intraresidual	161	151	156
interresidual	245	223	270
exchangeable	67	73	99
other restraints			
hydrogen bonds	82	82	82
dihedral angles	72	35	36
planarity	10	10	10
structural statistics:			
pairwise heavy atom RMSD value (Å)			
G-tetrad core	0.84 ± 0.18	0.91 ± 0.26	1.01 ± 0.17
all residues	2.70 ± 0.46	2.37 ± 0.36	2.64 ± 0.39
NOE violations (Å)			
maximum violation	0.144	0.197	0.366
mean NOE violation	0.0010 ± 0.0004	0.0016 ± 0.0005	0.0018 ± 0.0011
deviations from idealized geometry			
bond lengths (Å)	0.01 ± 0.0001	0.01 ± 0.0001	0.01 ± 0.0001
bond angles (degree)	2.20 ± 0.03	2.17 ± 0.03	2.19 ± 0.03

Table S5. List of ^{1}H chemical shifts (in ppm) of free *Myc-dup3*; only those protons are listed that were used for a chemical shift footprint.[a,b]

residue	imino	H6/H8	H2/H5/Me	H1’
T1	n.d.	7.20	1.64	5.76
G2	n.d.	7.61	-	5.57
A3	-	7.99	n.d.	5.82
G4	11.62	7.96	-	6.04
G5	11.22	7.59	-	6.08
G6	10.83	7.55	-	6.19
T7	n.d.	7.85	1.98	6.52
G8	11.62	7.96	-	6.13
G9	11.46	7.78	-	6.17
G10	11.31	7.79	-	6.36
T11	n.d.	7.67	1.94	6.25
A12	-	8.53	8.34	6.66
G13	11.83	8.08	-	6.16
G14	11.25	7.80	-	6.24
G15	11.12	7.79	-	6.41
T16	n.d.	7.84	1.98	6.50
G17	11.21	7.89	-	5.99
G18	11.25	7.88	-	6.10
G19	11.09	7.72	-	6.00
C20	-	7.56	5.43	6.07
T21	13.55	7.33	1.61	5.40
A22	-	8.25	7.31	6.02
G23	12.66	7.59	-	5.79
T24	13.64	7.16	1.24	5.91
C25	-	7.33	5.61	5.57
A26	-	8.16	n.d.	6.17
T27	n.d.	7.52	1.78	5.99
T28	n.d.	7.33	1.58	5.74
T29	n.d.	7.40	1.62	5.97
T30	n.d.	7.38	1.82	5.69
G31	12.65	7.92	-	5.54
A32	-	8.18	7.78	6.21

C33	-	7.18	5.14	5.71
T34	13.66	7.17	1.43	5.23
A35	-	7.85	7.45	5.92
G36	n.d.	7.28	-	5.47

[a] At 20 °C in 10 mM potassium phosphate buffer, pH 7. [b] n.d.: not determined.

Table S6. List of ^{1}H chemical shifts (in ppm) of *Myc-dup3* after addition of one equivalent PIQ; only those protons are listed that were used for a chemical shift footprint.[a,b]

residue	imino	H6/H8	H2/H5/Me	H1'
T1	n.d.	7.19	1.63	5.76
G2	n.d.	7.60	-	5.56
A3	-	7.95	-	5.81
G4	11.61	7.95	-	6.01
G5	11.08	7.58	-	6.07
G6	10.32	7.64	-	6.05
T7	n.d.	7.85	1.99	6.51
G8	11.61	7.91	-	6.08
G9	11.42	7.77	-	6.08
G10	11.00	7.83	-	6.38
T11	n.d.	7.65	1.94	6.24
A12	-	8.52	8.32	6.66
G13	11.80	8.07	-	6.14
G14	11.11	7.76	-	6.17
G15	11.06	7.76	-	6.40
T16	n.d.	7.85	1.99	6.50
G17	11.12	7.85	-	5.93
G18	11.06	7.82	-	6.09
G19	n.d.	7.52	-	6.08
C20	-	7.56	5.66	5.78
T21	n.d.	7.26	1.57	5.31
A22	-	8.20	n.d.	5.97
G23	12.65	7.53	-	5.74
T24	13.61	7.13	1.21	5.88
C25	-	7.32	5.59	5.57
A26	-	8.16	n.d.	6.17

T27	n.d.	7.52	1.78	5.99
T28	n.d.	7.32	1.58	5.72
T29	n.d.	7.40	1.63	5.97
T30	n.d	7.38	1.81	5.68
G31	12.64	7.91	-	5.54
A32	-	8.18	7.77	6.21
C33	-	7.19	5.12	5.71
T34	n.d.	7.19	1.45	5.23
A35	-	7.82	n.d.	5.77
G36	n.d.	7.30	-	5.50

[a] At 20 °C in 10 mM potassium phosphate buffer, pH 7. [b] n.d.: not determined.

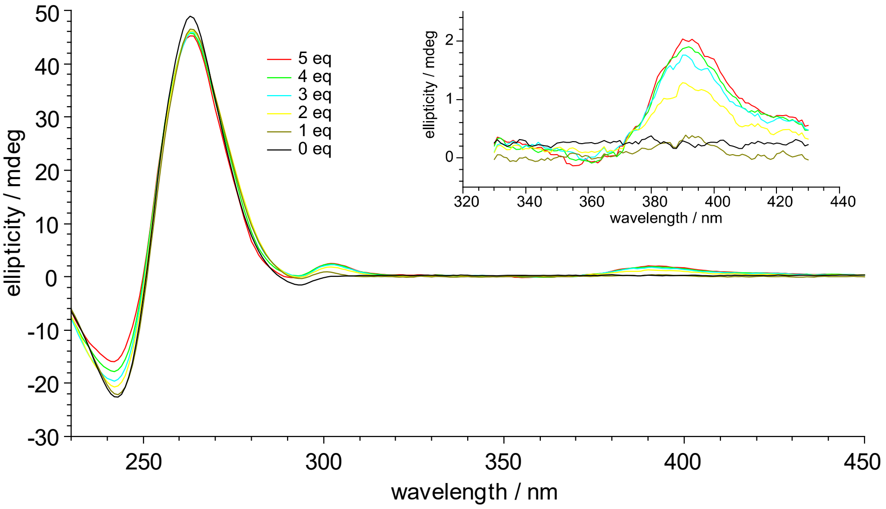

Figure S1. CD spectrum of *Myc* (5 μM) following titration with PIQ (0-5 equivalents) in 100 mM KCl, 20 mM potassium phosphate buffer, pH 7.0; the inset shows induced CD effects at the ligand absorption (from ref. 30).

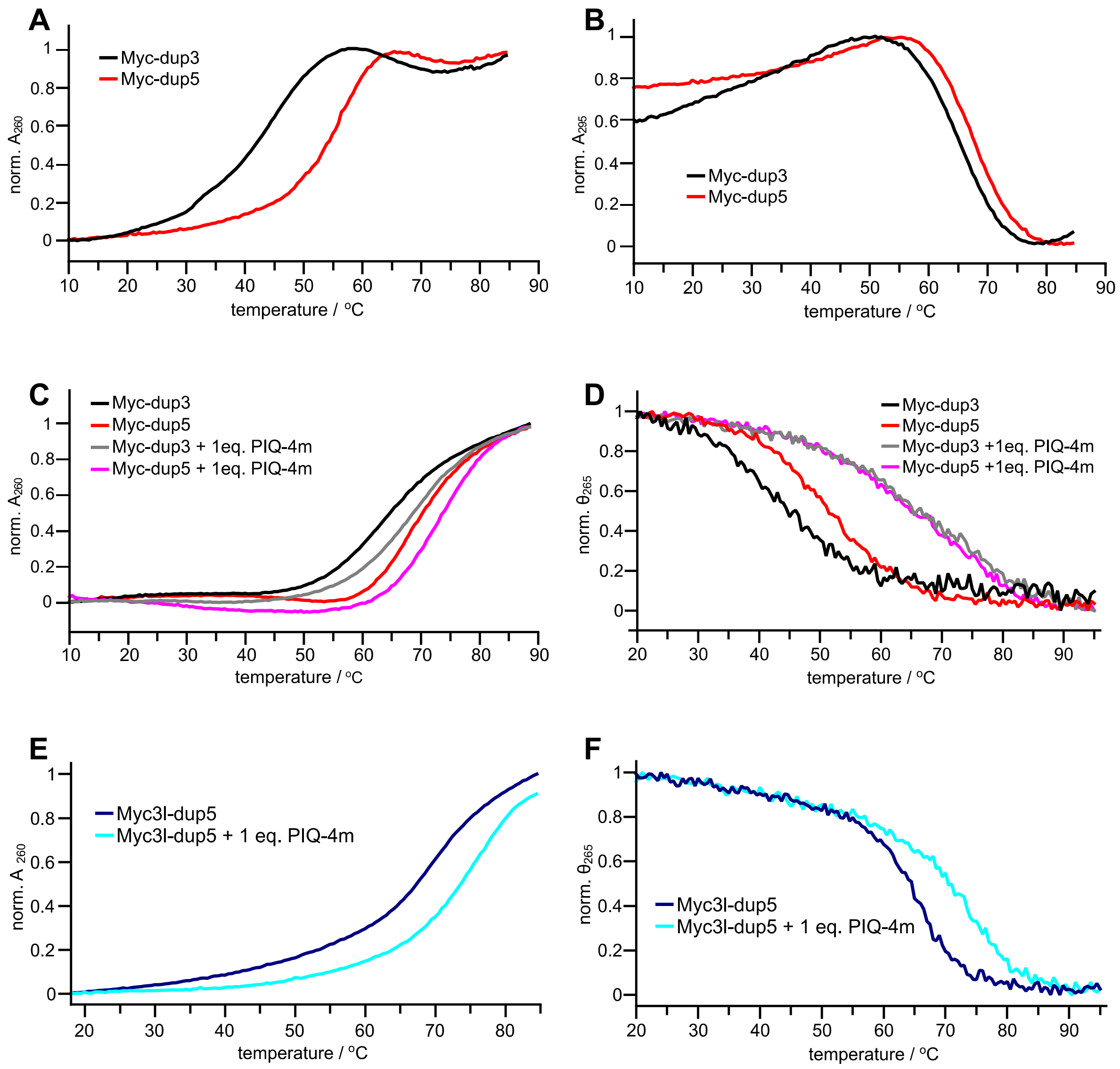

Figure S2. Representative optical melting curves of *Myc-dup3*, *Myc-dup5*, and *Myc3l-dup5*. (A) Normalized absorbances at 260 nm and (B) at 295 nm of *Myc-dup3* and *Myc-dup5* without ligand in 10 mM potassium phosphate buffer, pH 7. (C) Absorbance at 260 nm of *Myc-dup3* and *Myc-dup5* reflecting duplex melting without and with the addition of ligand in a 1:1 molar ratio in 100 mM NaCl, 20 mM sodium phosphate buffer, pH 7. (D) Ellipticity at 265 nm of *Myc-dup3* and *Myc-dup5* reflecting quadruplex melting without and with 1 equivalent of added ligand in 100 mM NaCl, 20 mM sodium phosphate buffer, pH 7. (E) Absorbance at 260 nm of *Myc3l-dup5* reflecting duplex melting without and with the addition of ligand in a 1:1 molar ratio in 100 mM NaCl, 20 mM sodium phosphate buffer, pH 7. (F) Ellipticity at 265 nm of *Myc3l-dup5* reflecting quadruplex melting without and with 1 equivalent of added ligand in 100 mM NaCl, 20 mM sodium phosphate buffer, pH 7.

Differential scanning calorimetry

DSC experiments were performed with a VP-DSC instrument (Malvern Instruments, United Kingdom). The oligonucleotide (50 µM) was dissolved in 10 mM potassium phosphate buffer, pH 7.0. The solution was heated with a heating rate of 0.5 °C/min. Data for a buffer versus buffer scan were subtracted from data obtained for a sample versus buffer scan. A cubic baseline was constructed and melting temperatures were determined from the peaks following deconvolution of the melting transitions. Data were analyzed with the Origin software.

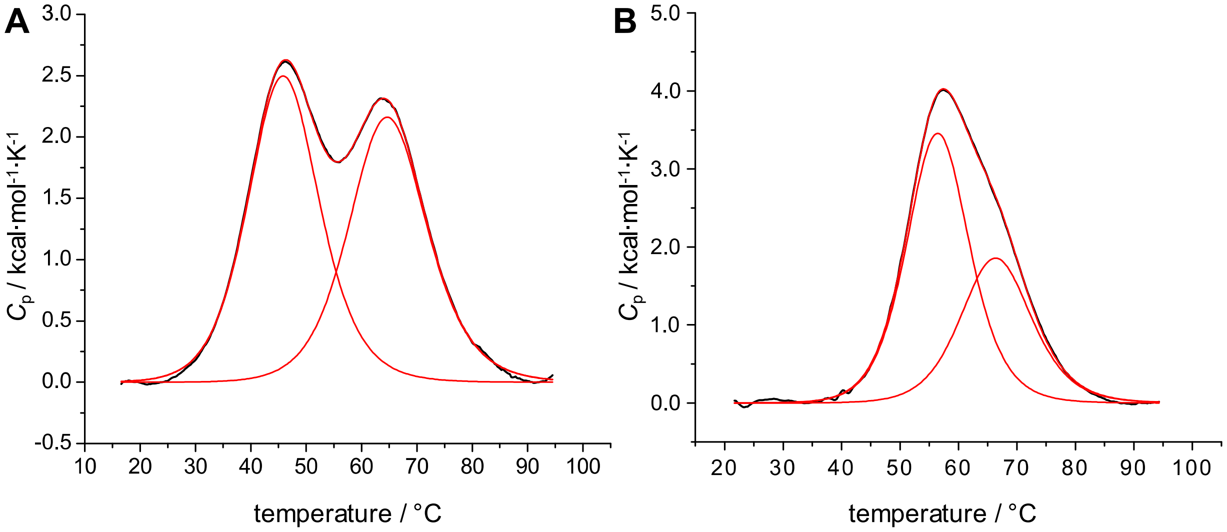

Figure S3. Representative DSC melting curves of (A) *Myc-dup3* and (B) *Myc-dup5* in 10 mM potassium phosphate buffer, pH 7. Thermograms were analyzed and fitted based on two transitions.

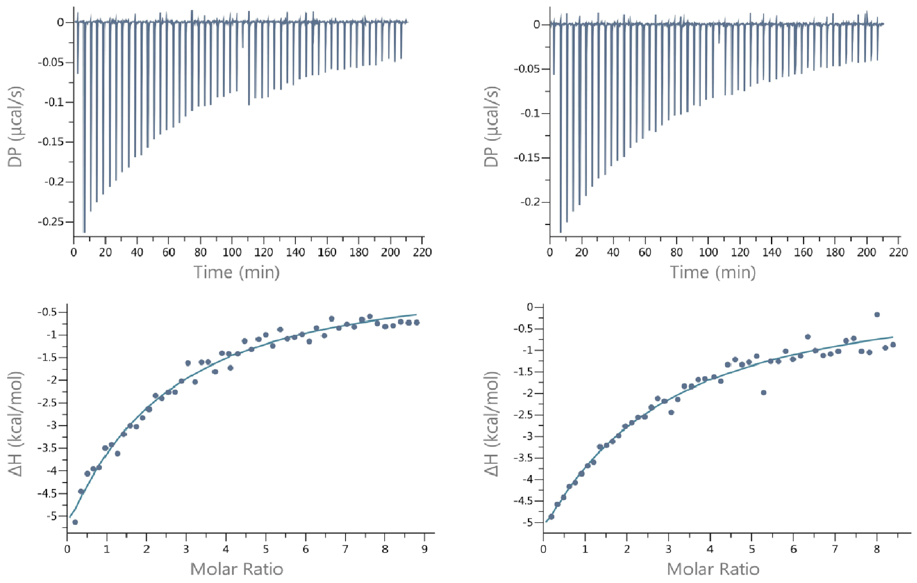

Figure S4. ITC thermograms of PIQ binding to duplex hairpins *Dup5* (left) and *Dup3* (right) at 40 °C. The upper and lower panel shows the heat burst for every injection step and the blank-corrected integrated heat versus molar ratio.

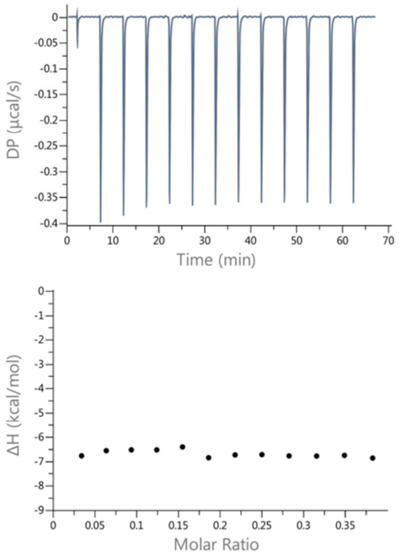

Figure S5. Representative excess-site ITC thermogram of PIQ titrated to *Myc-dup5* at 40 °C. The upper and lower panel shows the heat burst for every injection step and the blank-corrected integrated heat versus molar ratio.

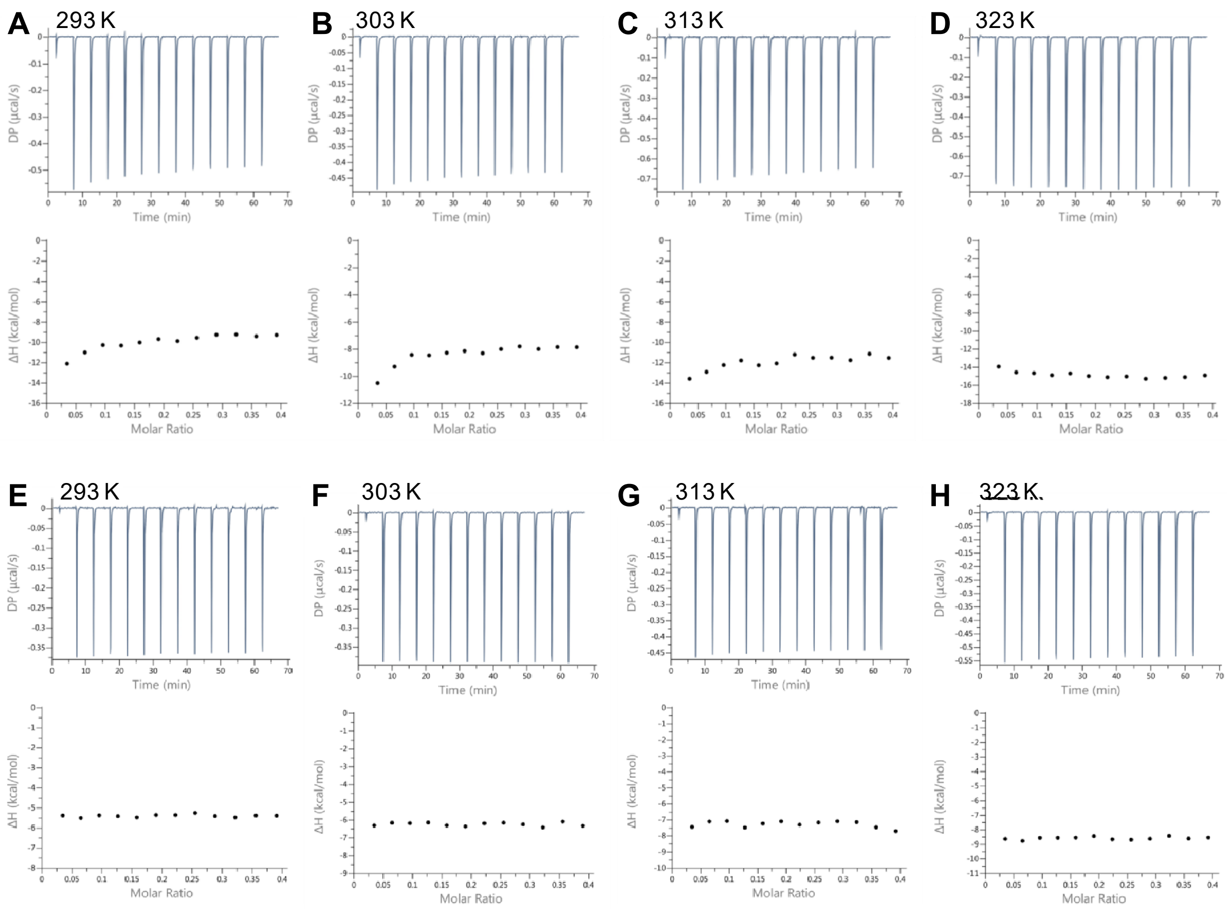

Figure S6. Representative excess-site ITC titrations of PIQ to *Myc-dup3* (A-D) and *Myc3l-dup5* (E-H) at different temperatures. The upper and lower panel shows the heat burst for every injection step and the blank-corrected integrated heat versus molar ratio.

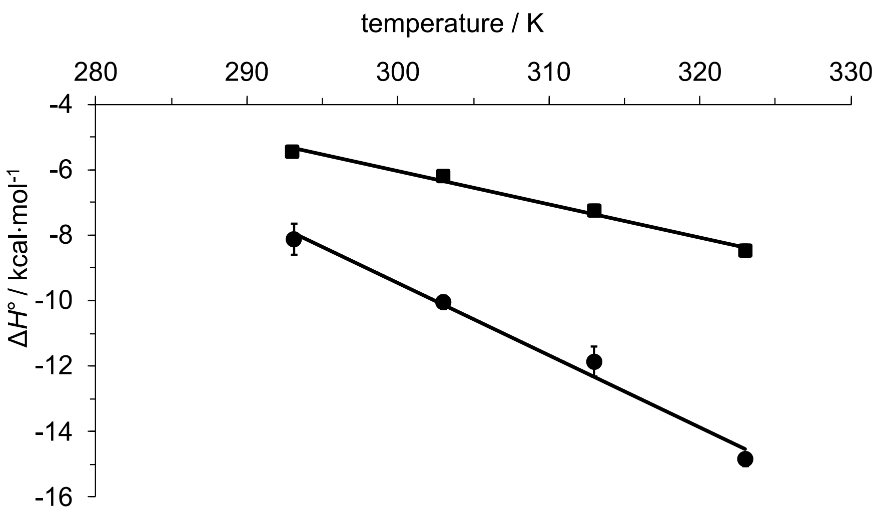

Figure S7. Plot of $\Delta H°$ as obtained from excess-site experiments for PIQ binding to *Myc-dup3* (circles) and *Myc3l-dup5* (squares) over temperature. $\Delta C_p°$ is given by the slope of the least squares regression line.

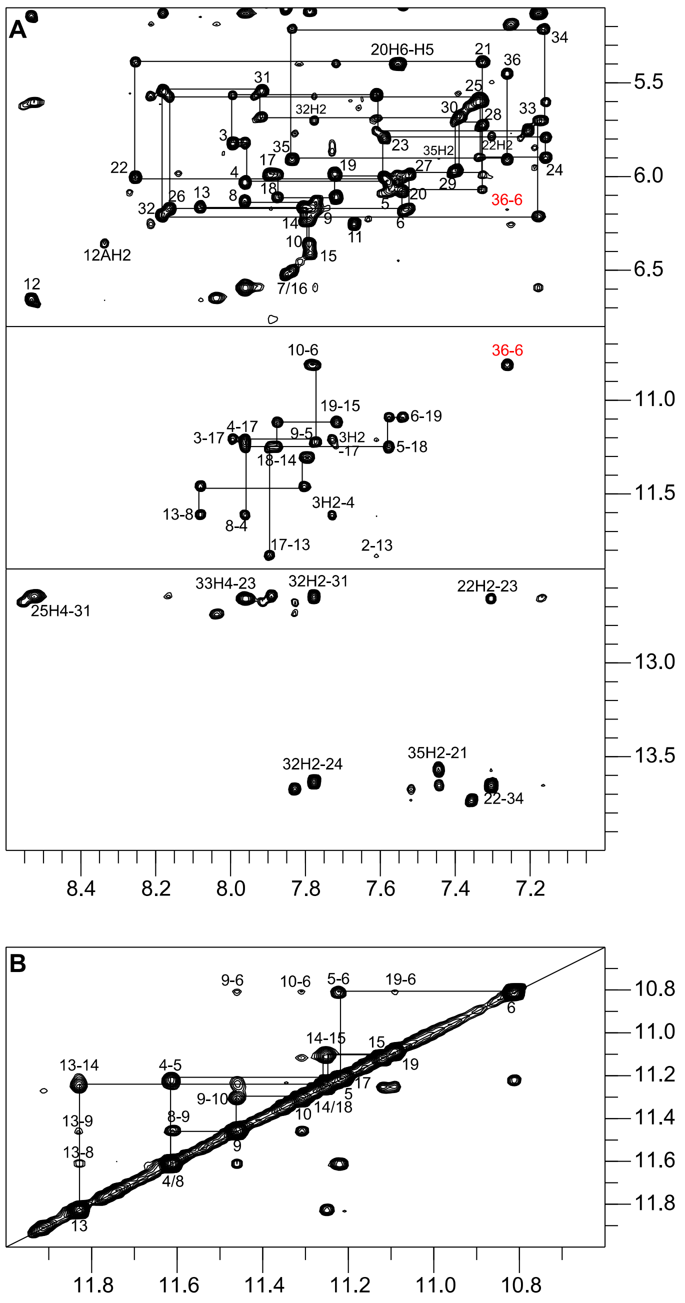

Figure S8. 2D NOESY spectral regions of *Myc-dup3*. (A) H6/H8(ω_2)-H1'(ω_1) (top), H6/H8(ω_2)-Hoogsteen imino(ω_1) (center), and AH2/CNH$_2$(ω_2)-WC imino(ω_1) connectivities (bottom). An uninterrupted NOE walk through H6/H8–H1' contacts can be followed from G17 along tract IV of the quadruplex to G36 at the duplex 3'-terminus (top); G36 H8 shows weak and strong crosspeaks (labeled in red) to G6 H1' (top) and to the G6 imino at the Q-D junction (center), respectively. (B) Imino-imino connectivities. Spectra were acquired with a 300 ms mixing time at 20 °C in 10 mM potassium phosphate buffer, pH 7.0.

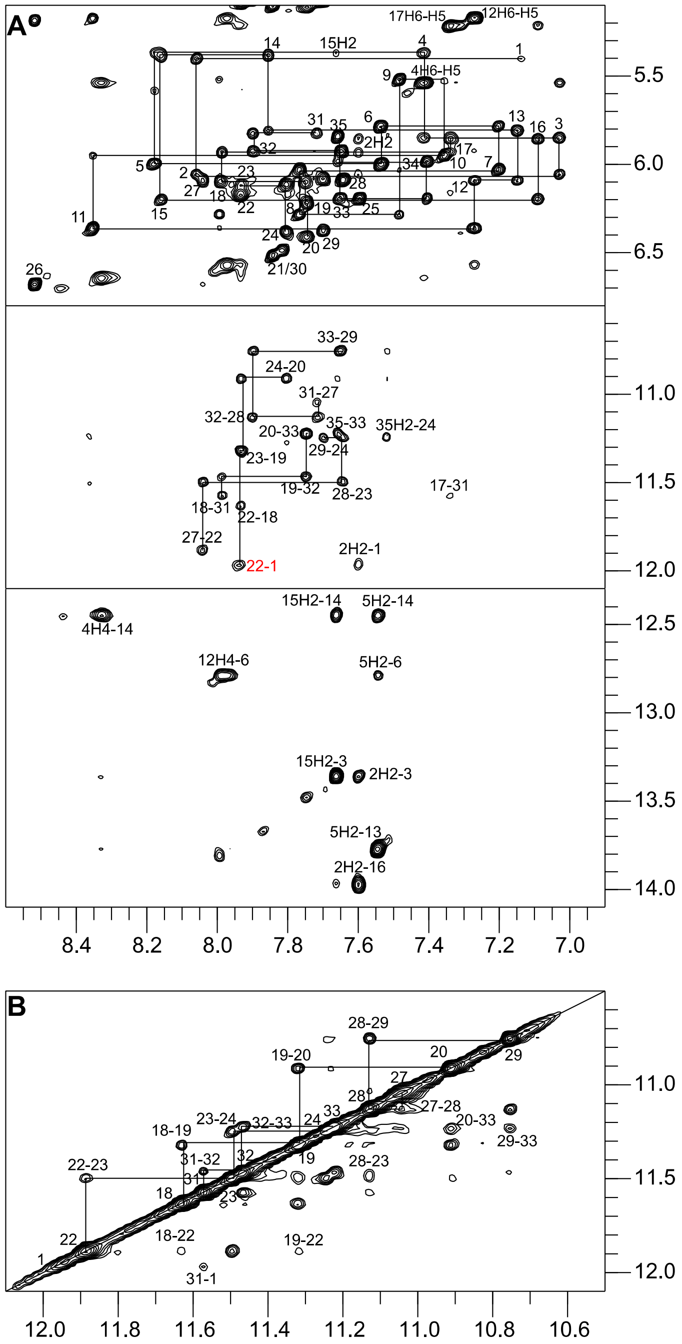

Figure S9. 2D NOESY spectral regions of *Myc-dup5*. (A) H6/H8(ω_2)-H1'(ω_1) (top), H6/H8(ω_2)-Hoogsteen imino(ω_1) (center), and AH2/CNH$_2$(ω_2)-WC imino(ω_1) connectivities (bottom). An uninterrupted sequential NOE walk through H6/H8–H1' contacts can be followed from G1 of the duplex along tract I of the quadruplex to G20 (top); G22 H8 shows a crosspeak to the G1 imino proton at the Q-D junction (center, labeled in red). (B) Imino-imino connectivities. Spectra were acquired with a 300 ms mixing time at 20 °C in 10 mM potassium phosphate buffer, pH 7.0.

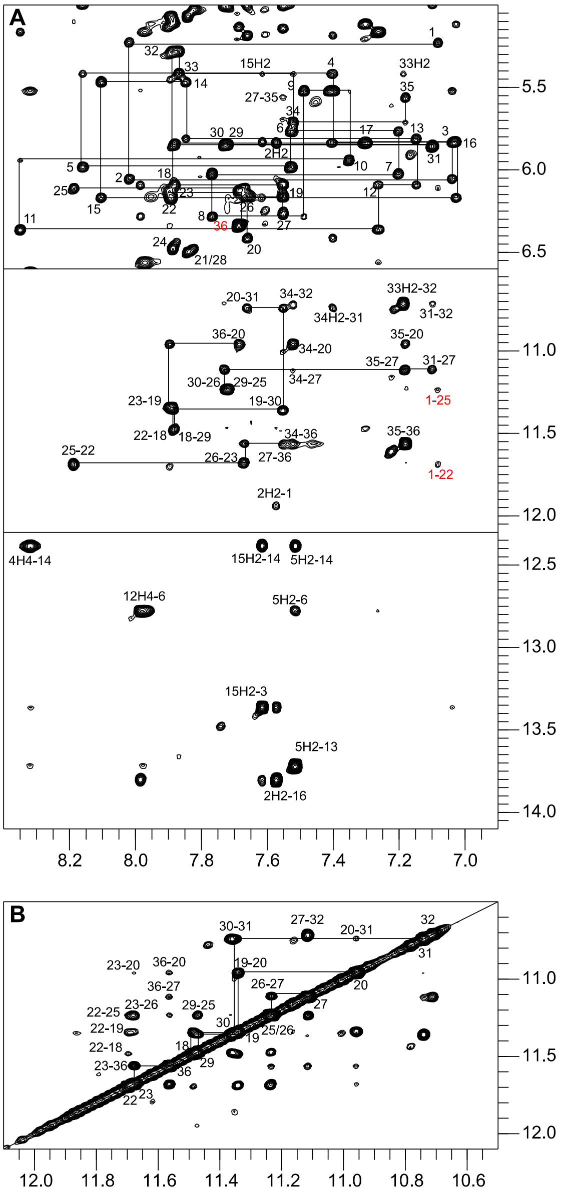

Figure S10. 2D NOESY spectral regions of *Myc3l-dup5*. (A) H6/H8(ω_2)-H1'(ω_1) (top), H6/H8(ω_2)-Hoogsteen imino(ω_1) (center), and AH2/CNH$_2$(ω_2)-WC imino(ω_1) connectivities (bottom). An uninterrupted sequential NOE walk through H6/H8–H1' contacts can be followed from G1 of the duplex along tract I of the quadruplex to G20 (top); a very strong H8-H1' intranucleotide crosspeak indicates a *syn* glycosidic torsion angle for G36 at the 3'-terminus (top, labeled in red); the G1 H8 proton shows crosspeaks to the imino protons of G22 and G25 at the Q-D junction (center, labeled in red). (B) Imino-imino connectivities. Spectra were acquired with a 300 ms mixing time at 20 °C in 10 mM potassium phosphate buffer, pH 7.0.

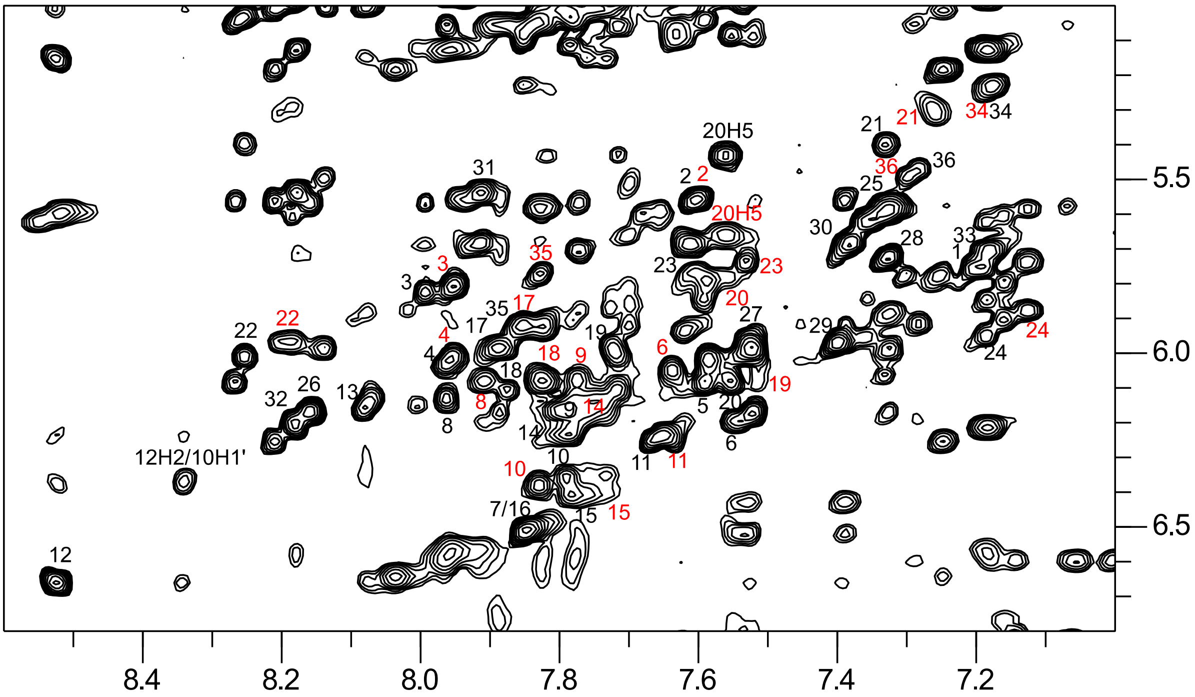

Figure S11. 2D NOESY spectrum of *Myc-dup3* (0.53 mM) with the addition of 0.5 equivalent of PIQ showing H6/8(ω_2)-H1'(ω_1) connectivities. Two sets of crosspeaks are observable with black and red assignments for crosspeaks of the free G4 and of the PIQ-G4 complex, respectively.

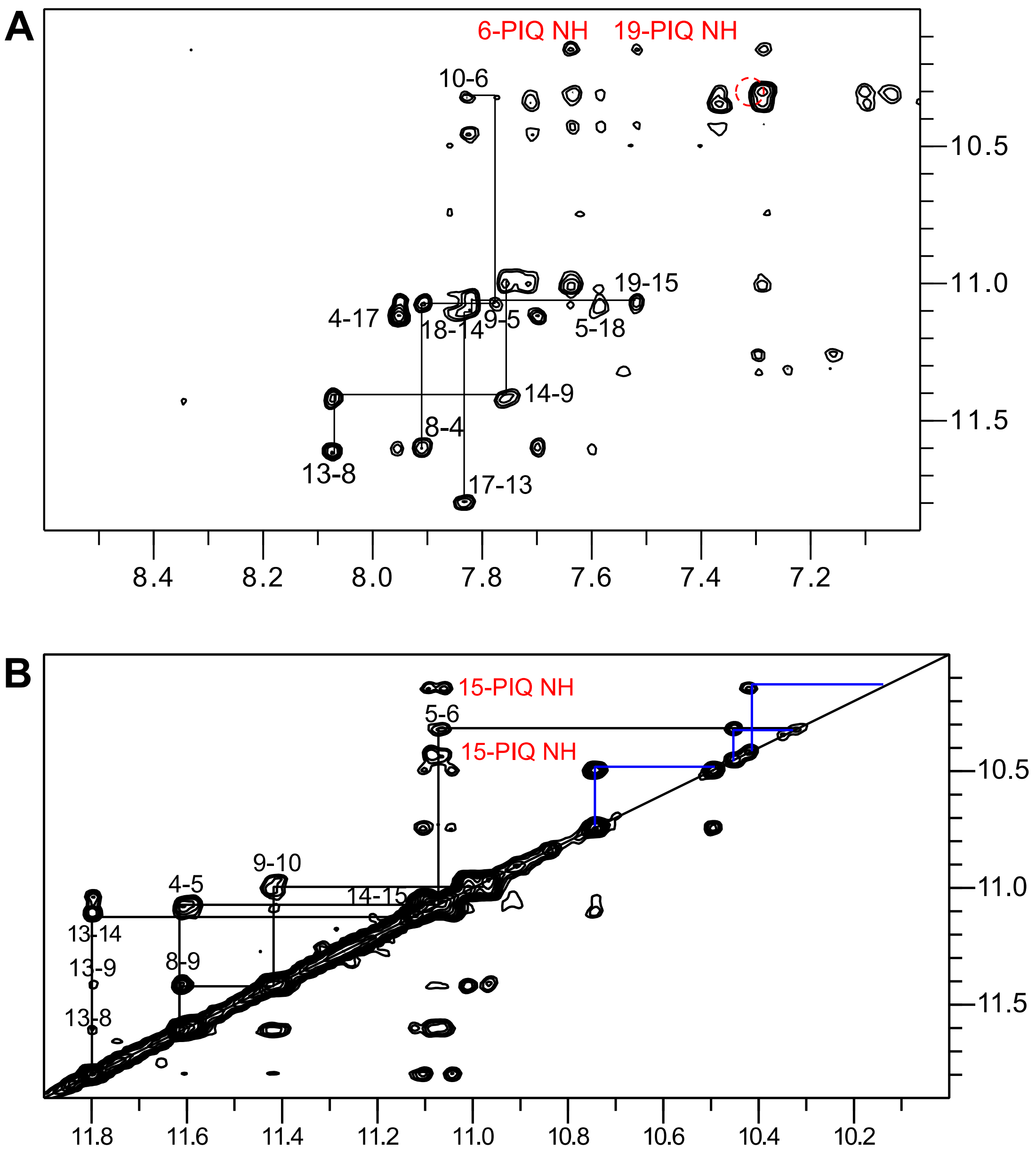

Figure S12. (A) H6/8(ω_2)-imino(ω_1) and (B) imino-imino 2D NOESY spectral region of *Myc-dup3* with addition of 1 equivalent of PIQ. The dashed red circle indicates the position of a missing contact between G36 and G6 at the junction as clearly observed in the free hybrid. Intermolecular crosspeaks with PIQ NH protons are labeled in red and exchange peaks of the ligand as demonstrated by additional ROESY spectra are indicated by blue lines. NOESY spectra were recorded with a 300 ms mixing time.

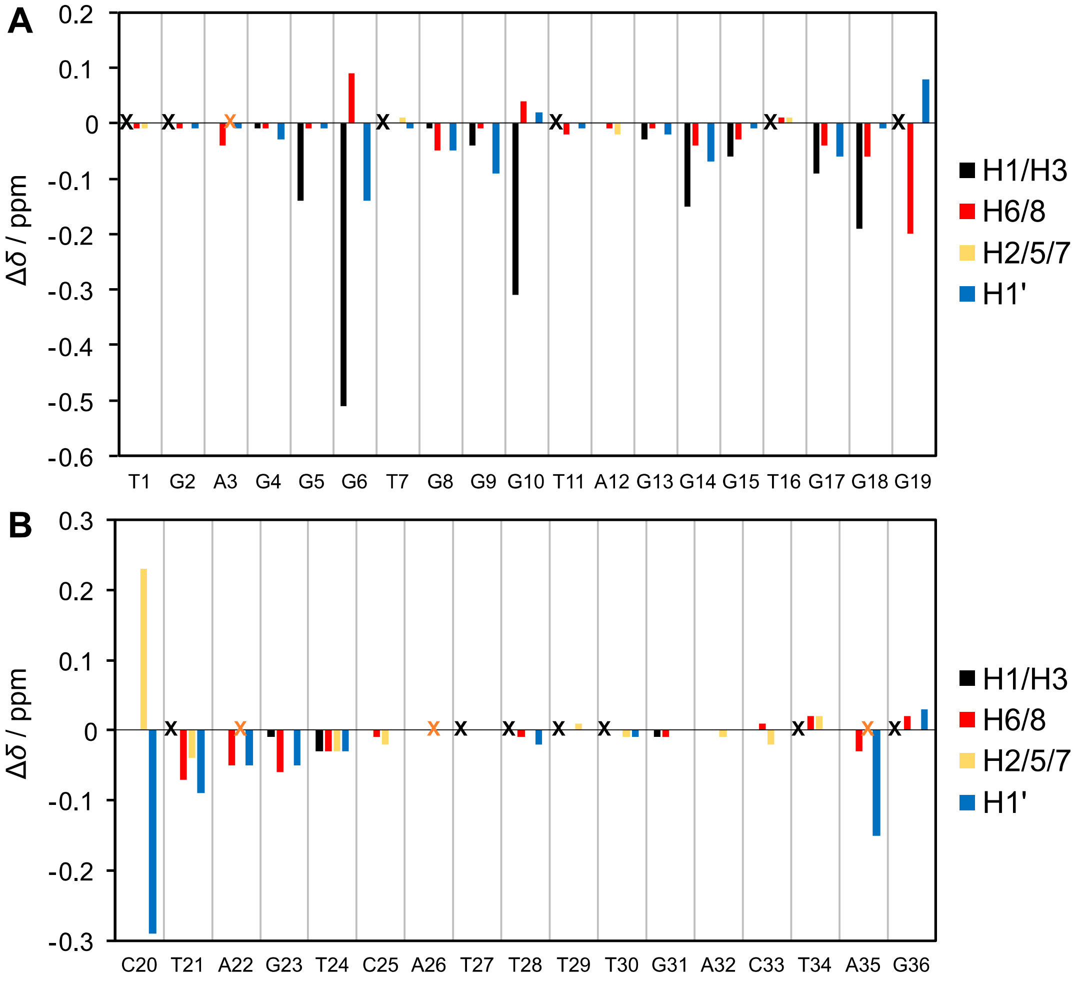

Figure S13. ^{1}H chemical shift differences for G4 residues in (A) the quadruplex subunit and (B) the duplex extension between complexed (with 1 eq. of PIQ) and free *Myc-dup3*; resonances marked by a cross could not be unambiguously assigned. For a more detailed compilation of chemical shift data see Tables S5 and S6.

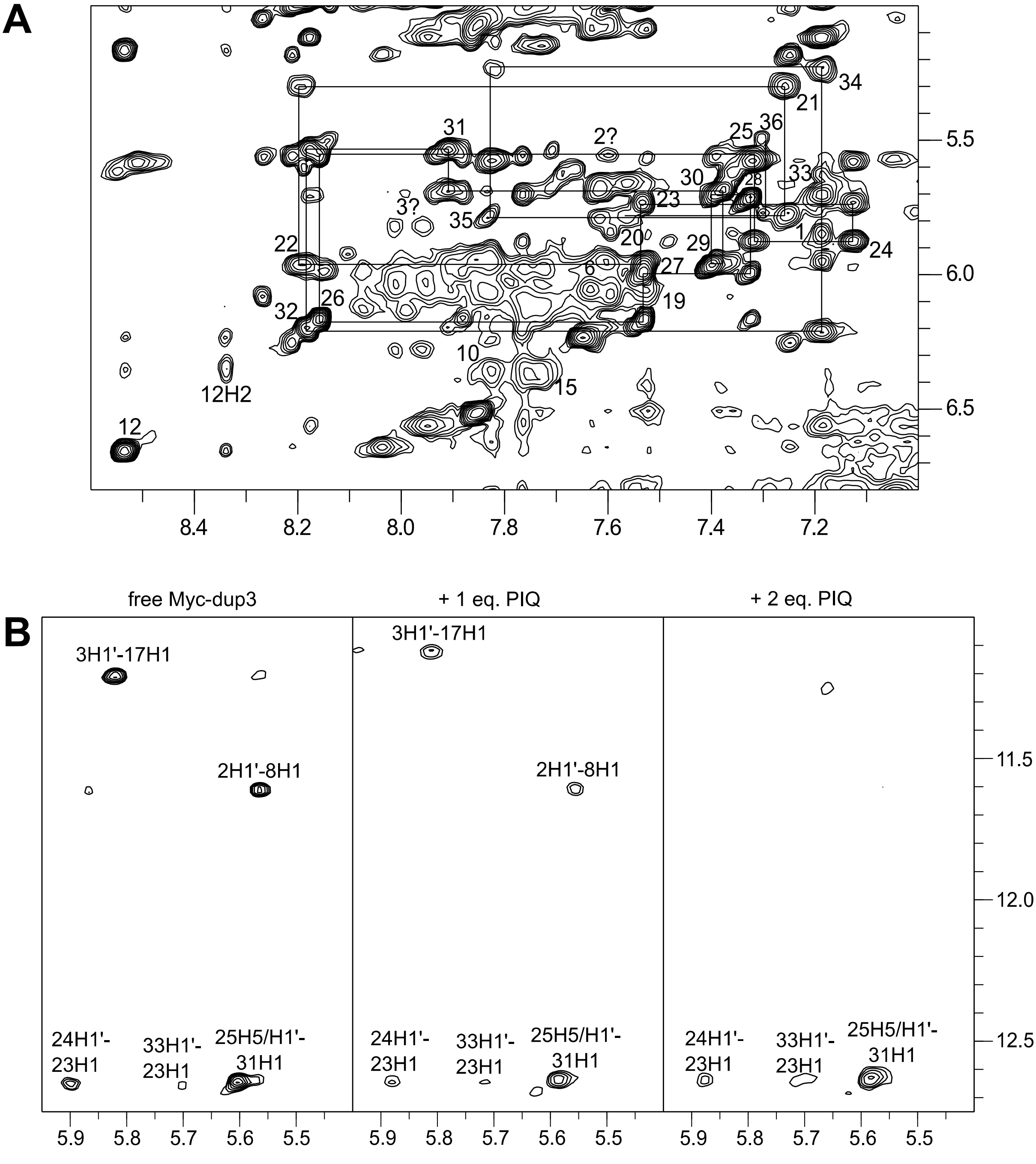

Figure S14. (A) H6/8(ω_2)-H1'(ω_1) 2D NOESY spectral region of *Myc-dup3* (0.53 mM) in the presence of 2 equivalents of PIQ; a continuous NOE walk is traced along the stem-loop duplex. (B) H1'(ω_2)-imino(ω_1) 2D NOESY spectral region of *Myc-dup3* in the absence (left) and in the presence of 1 equivalent (center) and 2 equivalents PIQ (right). All 2D NOESY spectra were acquired with a 300 ms mixing time at 20 °C in 10 mM potassium phosphate buffer, pH 7.0.

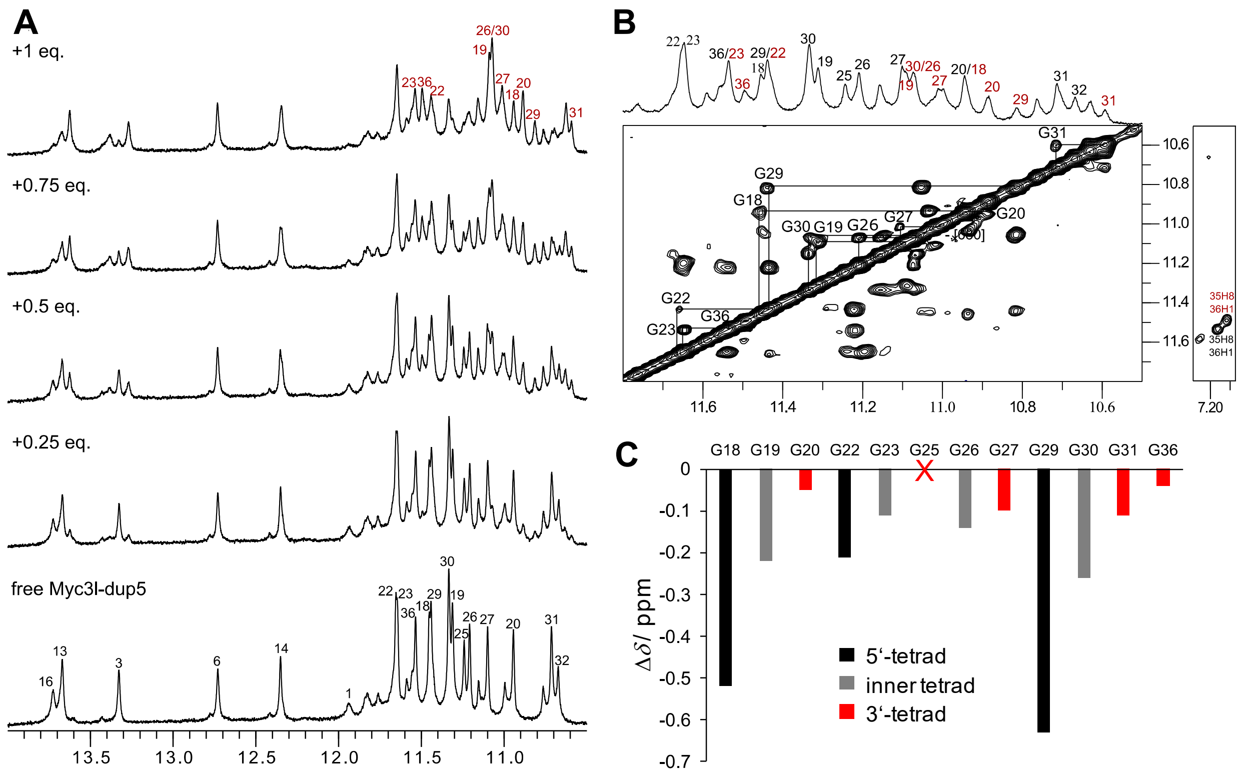

Figure S15. (A) Imino proton spectral region of *Myc3l-dup5* (0.62 mM) titrated with PIQ at 30 °C in 10 mM potassium phosphate buffer, pH 7.0. (B) 1D and ROESY spectrum showing the G4 imino proton spectral region of *Myc3l-dup5* in the presence of 0.5 equivalent of PIQ at 30 °C. Exchange crosspeaks connect guanine imino resonances of free and complexed species. A small shift of the G36 H1 resonance with a corresponding exchange peak close to the diagonal is confirmed by unambiguous G35 H8-G36 H1 NOE contacts (right). Imino proton resonances are assigned to G residues in free *Myc3l-dup5* and in the ligand-hybrid complex by black and red numbers, respectively. (C) Imino chemical shift differences of G4 residues between complexed (with 0.5 eq. of PIQ) and free *Myc3l-dup5*. The imino resonance of G25 could not be unambiguously assigned.

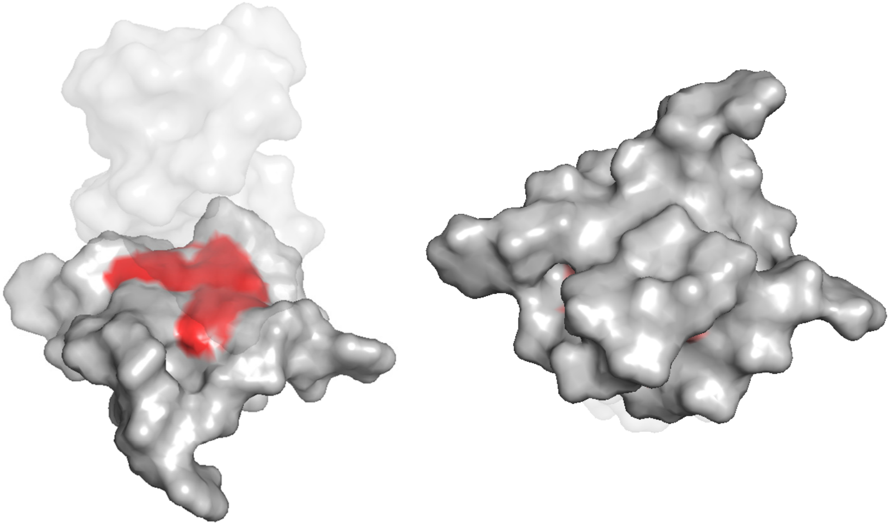

Figure S16. Guanosine imino proton chemical shift perturbations of the quadruplex after addition of 0.6 equivalent of PIQ mapped with red color of variable intensity on a surface model of the *Myc-dup5* hybrid. View onto the 5'-tetrad of the quadruplex-duplex junction (left) and onto the 3'-tetrad (right). The duplex extension is shown in a transparent representation.
